# Genome-wide association study reveals a set of genes associated with resistance to the Mediterranean corn borer (*Sesamia nonagrioides* L.) in a maize diversity panel

**DOI:** 10.1186/s12870-014-0403-3

**Published:** 2015-02-05

**Authors:** Luis Fernando Samayoa, Rosa Ana Malvar, Bode A Olukolu, James B Holland, Ana Butrón

**Affiliations:** Misión Biológica de Galicia, Spanish National Research Council (CSIC), P.O. Box 28, 36080 Pontevedra, Spain; Department of Crop Science, North Carolina State University, Raleigh, North Carolina 27695 USA; U.S. Department of Agriculture-Agricultural Research Service, Plant Science Research Unit, Raleigh, North Carolina 27695 USA

**Keywords:** Candidate genes, Corn borer, Genome-wide association study, Insect resistance, Maize, Mixed linear models, *Sesamia nonagrioides*

## Abstract

**Background:**

Corn borers are the primary maize pest; their feeding on the pith results in stem damage and yield losses. In this study, we performed a genome-wide association study (GWAS) to identify SNPs associated with resistance to Mediterranean corn borer in a maize diversity panel using a set of more than 240,000 SNPs.

**Results:**

Twenty five SNPs were significantly associated with three resistance traits: 10 were significantly associated with tunnel length, 4 with stem damage, and 11 with kernel resistance. Allelic variation at each significant SNP was associated with from 6 to 9% of the phenotypic variance. A set of genes containing or physically close to these SNPs are proposed as candidate genes for borer resistance, supported by their involvement in plant defense-related mechanisms in previously published evidence. The linkage disequilibrium decayed (*r*^2^ < 0.10) rapidly within short distance, suggesting high resolution of GWAS associations.

**Conclusions:**

Most of the candidate genes found in this study are part of signaling pathways, others act as regulator of expression under biotic stress condition, and a few genes are encoding enzymes with antibiotic effect against insects such as the *cystatin1* gene and the defensin proteins. These findings contribute to the understanding the complex relationship between plant-insect interactions.

**Electronic supplementary material:**

The online version of this article (doi:10.1186/s12870-014-0403-3) contains supplementary material, which is available to authorized users.

## Background

Corn borers are the primary maize pest in many environments [1,2]. Corn borers feeding on the pith of the stem results in yield losses because stem damage interferes with assimilate movement to developing kernels. They can also attack the ears, promoting secondary fungal infection, leading to contamination of grain with mycotoxins that may affect human and animal health [3,4].

There are different species of borers that attack maize in different parts of the world. The most economically important species are classified into two families: Crambidae and Noctuidae. Within the Crambidae family, the species with economic importance are: *Ostrinia nubilalis* Hubner in North America, Europe and North Africa; *Ostrinia furnacalis* Guenée in Asia; *Diatraea saccharalis* Fabricius from USA to Argentina; *Chilo partellus* Swinhoe in Southern USA, Central America and the Caribbean; *Diatraea lineolata* Walker in Central America, the Caribbean region and South America; and *Diatraea grandiosella* Dyar in North and Central America. Within the Noctuidae family, the main maize borers are: *Sesamia nonagrioides* Lefebvre in the Mediterranean region, *Busseola fusca* Fuller in sub-Saharan Africa, and *Sesamia calamistis* Hampson in West Africa. This study focuses on the noctuid Mediterranean corn borer (MCB) *Sesamia nonagroides* Lefebvre, the most important insect pest of maize in the Mediterranean region that includes Southern Europe [2,5,6].

The use of transgenic corn which produces *Bacillus thutingiensis* (*Bt*) toxins is a good method for controlling these pests, but transgenic crops are not authorized in several European countries under any agricultural system [7] and are not allowed for organic production [8]. In addition, recent studies have reported a reduction of efficacy of *Bt* transgenes caused by evolved resistance of some important pests [9-11]. The stacking of several resistant genes has been proposed as one means to delay insect adaptation [12]. Natural sources of resistance to stem borers in maize could reveal promising genes for use in either breeding or transgenic approaches to resistance.

In Spain, there are three MCB generations per year and the second and subsequent generations are able of making significant damage on the stem and, secondarily, on the ear. Following artificial infestation, the level of maize host resistance to stem borer is measured by the tunnel length made by the larvae in the stem as well as by a visual scale for kernel damage. These traits have a complex genetic architecture because resistance depends on the plant-insect relationship, which is influenced by environmental conditions and the developmental stage of the host plant [13,14]. The line mean heritability estimates for tunnel length under corn borer infestation ranged among studies from moderate to relatively high (*h*^2^ = 0.50 to 0.78) [15-18], depending on the genetic background.

At present, commercial materials with high levels of native resistance to these insects are not available even though breeding for increasing maize resistance to corn borers has been conducted during the last three decades in different regions around the world. Klenke et al. [19] reduced tunnel length by 4 and 6 cm after four cycles of recurrent selection for resistance to the first and second generations of ECB, respectively. Bosque-Pérez et al. [20] described successful results in the development of materials with resistance to *S. calamistis* and African sugarcane borer (*Eldana saccharina* Walker). Sandoya et al. [21] achieved a reduction of 1.8 cm per cycle of recurrent selection for tunnel length by MCB. In addition, a negative relationship between resistance to stem borers and yield has been found [22] when selection for improved yield under infestation was practiced [23]. In summary, classical breeding experiments have demonstrated some successful improvement in corn borer resistance, but natural levels of resistance in elite cultivars remain insufficient to manage the pest. Detection of stem borer resistance QTL could enhance breeding for this trait via marker-assisted breeding or genomic selection.

Genetic effects for resistance against borer attack fit an additive – dominant model, although additive effects appear to be the most important in determining resistance to tunnel length and kernel damage [14,24-28]. Therefore, the study of genetic factors involved in maize resistance to borers can be performed using highly inbred lines.

Several studies performed with segregating biparental populations have reported genomic regions containing minor and major quantitative traits loci (QTL) for resistance to stem and leaf attack by European corn borer (ECB; [15-17,29-31]. Fewer studies have reported QTLs for resistance to other borer species such as the Mediterranean corn borer (MCB) [18,32] or some tropical borers [33-35]. In maize, QTLs for resistance to tunneling by corn borers have been detected on all chromosomes, with the most commonly detected regions occurring on chromosomes 1, 2, 3, 5, and 9 [1]. Identification of causal genes underlying the QTL for resistance in these genomic regions could help breeders to transfer the allelic variants that confer resistance from the lines that carry them to elite breeding lines that lack these resistance alleles. The identification of the causal genetic variants or markers in high LD with causal variants in diverse materials would minimize the risk of dragging other genes with negative effect on the agronomic value during the transfer process.

Although conventional QTL mapping based on linkage maps of biparental populations has been an efficient approach to detect regions related to the resistance to corn borers, higher resolution is needed to detect the genes involved in the defense mechanism of the plant. Genome wide association study (GWAS) based on linked disequilibrium (LD) in diverse genetic samples is a relatively new approach which offers higher resolution mapping that under optimal conditions can pinpoint causal genes underlying quantitative trait variation. Exploiting advances in genotyping and sequencing technology, this approach has been successful in detecting genes associated with diseases in humans [36-38], animals [39-42] and different quantitative traits in plants [43-46]. In contrast to the conventional QTL mapping approach based on linkage in a biparental population, GWAS is based on LD among extant lines from different populations, such that a large number of markers covering the whole genome are required [45,47]. In diverse maize samples, LD is low, therefore, many more markers are needed than in autogamous species (with higher LD) to adequately explore the genetic architecture of complex traits [48]. The low LD offers the benefit of better resolution to delineate potential causal genes within small LD blocks.

Many single-nucleotide polymorphism (SNP) markers have been identified and scored on a maize diversity panel (composed of 302 inbred line) that represents the diversity available in public breeding sector around the world [49,50]. The population has been successfully used by the maize community to perform GWAS in economically important quantitative traits such as kernel composition [51], hypersensitive response [52] and *Fusarium* ear rot resistance [53].

To date, no GWAS for insect resistance in maize has been reported, although a few GWA studies that deal with plant defense mechanisms against insect attack have been reported in other plant species [54,55]. In this study, we performed GWAS to identify SNPs associated with resistance to MCB. GWAS was done in a subset (270 inbreds) of the maize diversity panel using the maize 50 k SNP genotyping array [56] plus a more recently developed set of 425 k SNPs found through genotyping by sequencing [57,58].

## Results

### Means, analysis of variance and heritabilities

Differences among inbred lines were highly significant (*P* < 0.01) for all resistance (TL, SD, and KR) and agronomic traits (PH, DTA, and DTS); while significant (*P* < 0.05) genotype by environment (G × E) interactions were observed for all traits as shown in Additional file 1: Table S1 and S2. The inbred means for TL ranged from 5.2 to 49.2 cm with an overall mean of 20.9 cm, and for SD from 4.1 to 22.6% with an overall mean of 11.9%, respectively. The inbred mean for KR ranged from 5.4 to 9 with an overall mean of 7.8 in the subjective scale. Higher values for TL were observed in 2012 (overall mean = 43.3 cm) compared with 2010 (overall mean = 17.5 cm) and 2011 (overall mean = 14.7 cm, Additional file 1: Table S3 and S4). Intermediate values for heritability on a line mean-basis were estimated for TL (*h*^2^ = 0.60) and KR (*h*^2^ = 0.52) across the three years, whereas a low heritability value was obtained for SD (*h*^2^ = 0.25, Additional file 1: Table S1).

### Correlation analysis

A significant and high (*r* > 0.50) phenotypic correlation was observed between TL and SD. Genetic correlation coefficients were also significant and high between TL and SD and between TL and PH (Table 1). KR showed significant phenotypic correlations with other resistance traits but the correlation coefficients were not higher than 0.5. Genetic correlations between KR and the agronomic traits (DTA, DTS and PH) were significant and high (Table 1).

**Table 1:** **Genotypic** ^**1**^ **(above diagonal) and phenotypic** ^**2**^ **(below diagonal) correlation coefficient estimates for each pair of traits**

	**TL**	**SD**	**KR**	**DTA**	**DTS**	**PH**
TL		0.97*	NS	NS	NS	0.51*
SD	0.74*		NS	NS	NS	NS
KR	−0.31*	−0.31*		0.75*	0.71*	0.51*
DTA	NS	NS	0.30*		0.99*	0.66*
DTS	NS	NS	0.28*	0.96*		0.65*
PH	0.24*	−0.06	0.24*	0.51*	0.48*	

TL, tunnel length; SD, stem damage; KR, kernel resistance; PH, plant height; DTA, days to anthesis; DTS, days to silking.

^1^ Genotypic correlation coefficients were considered significant, *, when exceeded twice its standard error; NS, not significant.

^2^ Phenotypic correlation coefficients were considered significant, *, at 0.05 probability level according to Steel and Torrie [59]; NS, not significant.

### Association analysis of maize resistance to MCB and agronomic traits

The compressed mixed linear model computed for each trait in Tassel reduced the pairwise kinship matrix by clustering the 267 lines into 32 groups for TL, 201 groups for SD, 166 groups for KR, 192 for PH, and 240 for DTA, and DTS (Table 2). The proportion of the total phenotypic variation explained by background genetic effects was 39, 13, and 48% for TL, SD, and KR, respectively. By comparison, the background polygenic effects modeled by the K matrix accounted for 60, 94 and 90% of total variation for PH, DTA, and DTS, respectively.

**Table 2:** **Summary of the compressed mixed linear model analysis for three traits related to resistance to MCB attack and three agronomic traits in an inbred association panel evaluated in three years**

**Trait**	***n*** ^**a**^	***s*** ^**b**^	**Compression Level (** ***c*** **)** ^**c**^	$\left({\widehat{\boldsymbol{\upsigma}}}_{\mathbf{g}}^{\mathbf{2}}\right)$ ^**d**^	$\left({\widehat{\boldsymbol{\upsigma}}}^{\mathbf{2}}\right)$ ^**e**^	$\left(\frac{{\widehat{\boldsymbol{\upsigma}}}_{\mathbf{g}}^{\mathbf{2}}}{{\widehat{\boldsymbol{\upsigma}}}_{\mathbf{g}}^{\mathbf{2}} + {\widehat{\boldsymbol{\upsigma}}}^{\mathbf{2}}}\right)$ ^**f**^
TL	267	32	8.34	32.80	50.61	0.39
SD	267	201	1.33	1.45	9.51	0.13
KR	265	166	1.60	0.15	0.16	0.48
PH	266	192	1.39	384.70	256.56	0.60
DTA	266	240	1.11	45.03	5.03	0.94
DTS	266	240	1.11	51.19	5.87	0.90

TL, tunnel length; SD, stem damage; KR, borer kernel resistance; PH, plant height; DTA, days to anthesis; DTS, days to silking.

^a^ Total number of inbred lines included in the analysis.

^b^ Number of groups obtained using a clustering approach based on K matrix with the optimum compression level option.

^c^ Compression level is the average number of inbred lines per group estimated as n/s.

^d^ Additive background genetic variance component estimated in Tassel by fitting the K matrix in the MLM without any SNP marker effects.

^e^ Residual genotypic variance component estimated in Tassel.

^f^ Proportion of phenotypic variance explained by the K matrix, estimated as background genetic variance divided by total phenotypic variance.

**Figure 1:**
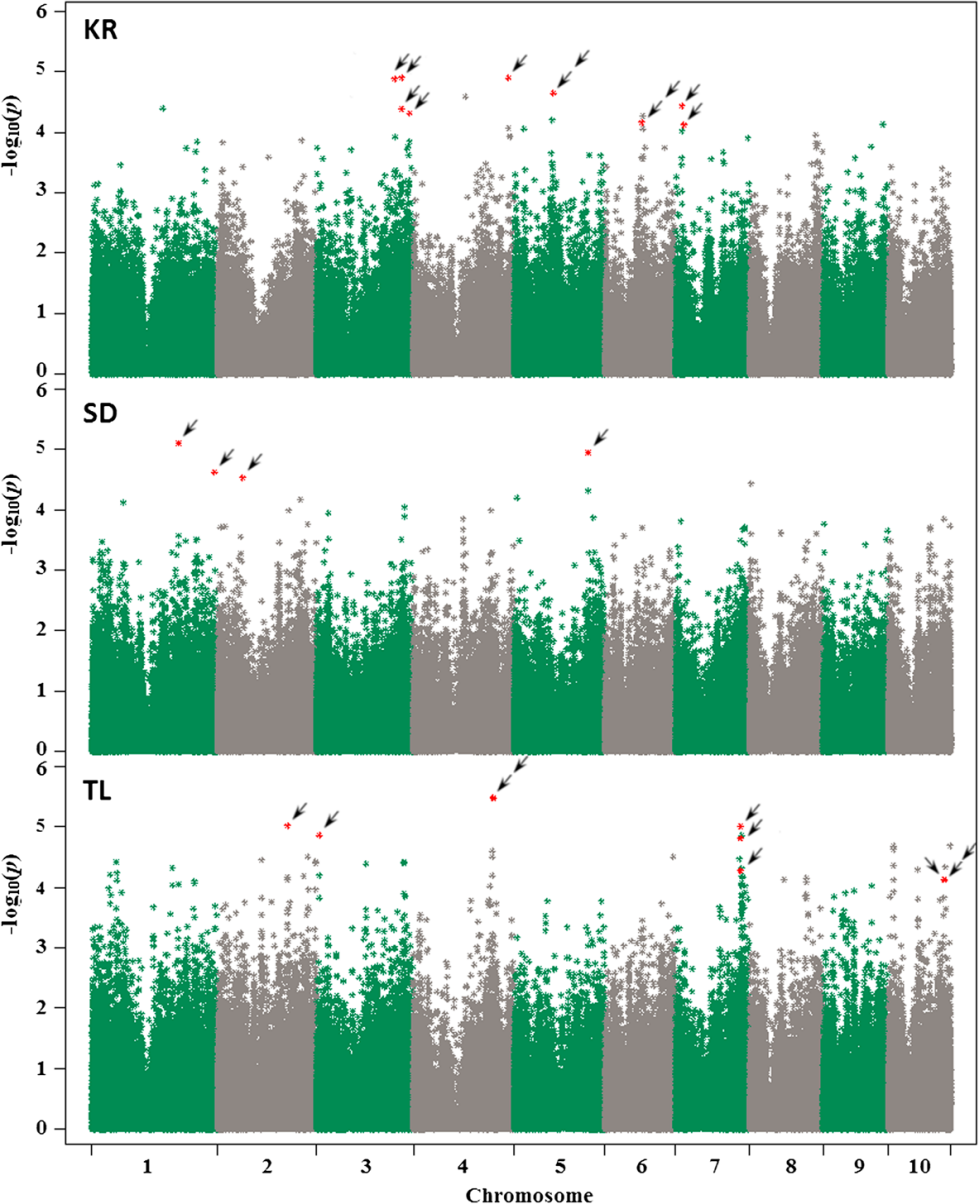
**GWAS results for the three resistance traits to MCB attack in a maize association panel.** Each graph (TL, SD, and KR) represent the *P*-values of the 246,477 SNPs tested for each resistance trait. Each row indicates the SNP significantly associated (RMIP ≥ 0.30) to each resistance trait analyzed.

Ten SNPs were identified as significantly associated with length of tunnels made by MCB (Figure 1, Table 3). Based on the additive effects, the major allele reduce TL for all significant SNPs except for the SNP located on chromosome 10 (Table 3). Four SNPs were significantly associated with SD made by MCB (Figure 1). The minor allele for those SNPs increases the SD from 1.1 to 2%. The total variance explained (*R*^*2*^) by each SNP associated with TL and SD ranged from 7 to 9%. Eleven SNPs were significantly associated with KR (Figure 1) and the total variance (*R*^*2*^) explained by each of them ranged from 6 to 8%. The minor allele for those SNP reduced from 0.15 to 0.40 points the ratio that accounts for kernel resistance.

**Table 3:** **SNP identification (SNP ID), additive effect and allelic variants for the SNP, proportion of total variance explained by the SNPs significantly associated with resistance traits (TL, SD, and KR), and significance values for the association between the SNP and the phenotype (** ***P*** **-value and RMIP)**

**Trait** ^**a**^	**SNP ID** ^**b**^	**Alleles** ^**c**^	**(N** ^**o**^ **)** ^**d**^	**Additive effect** ^**e**^	***P*** **-value**	**(** ***R*** ^**2**^ **)** ^**f**^	**RMIP** ^**g**^
TL	S2_168004182	G/T	225/36	3.09	9.51 × 10^−6^	0.08	0.51
TL	S3_7081859	T/A	207/55	2.52	1.38 × 10^−5^	0.07	0.50
TL	S4_190444179	G/A	218/38	3.18	3.26 × 10^−6^	0.09	0.37
TL	ss4_190679094^h^	G/A	226/41	3.09	3.37 × 10^−6^	0.08	0.45
TL	S7_154739818	G/C	248/14	4.25	5.28 × 10^−5^	0.07	0.37
TL	S7_154741622	C/T	217/47	2.84	9.84 × 10^−6^	0.08	0.56
TL	S7_155702328	T/A	171/67	2.43	1.55 × 10^−5^	0.08	0.38
TL	S10_133337924	G/T	133/99	−1.30	7.40 × 10^−5^	0.07	0.30
TL	S10_133337925	G/C	133/99	−1.30	7.40 × 10^−5^	0.07	0.30
TL	S10_133337950	T/C	133/99	−1.30	7.40 × 10^−5^	0.07	0.30
SD	S1_208315891	C/A	126/106	1.09	7.86 × 10^−6^	0.09	0.63
SD	S1_293163491	T/A	207/37	1.37	2.39 × 10^−5^	0.08	0.38
SD	S2_59729532	A/T	235/17	1.90	2.93 × 10^−5^	0.07	0.32
SD	ss5_176870721^h^	G/A	228/38	1.37	1.12 × 10^−5^	0.08	0.41
KR	S3_187742562	C/A	244/12	−0.38	1.30 × 10^−5^	0.08	0.48
KR	S3_204458505	T/A	186/65	−0.19	4.14 × 10^−5^	0.07	0.40
KR	S3_204586960	C/G	251/13	−0.37	1.25 × 10^−5^	0.08	0.44
KR	S3_222733400	C/T	142/110	−0.15	4.90 × 10^−5^	0.07	0.34
KR	S4_227101950	C/T	242/17	−0.30	1.26 × 10^−5^	0.08	0.43
KR	S4_227101985	A/T	242/17	−0.30	1.26 × 10^−5^	0.08	0.43
KR	S5_93580059	C/T	230/28	−0.24	2.27 × 10^−5^	0.07	0.31
KR	S6_88149024	A/G	187/55	−0.17	6.83 × 10^−5^	0.07	0.48
KR	S6_88149036	G/C	187/55	−0.17	6.83 × 10^−5^	0.07	0.48
KR	S7_15072370	G/A	230/24	−0.26	3.65 × 10^−5^	0.07	0.46
KR	S7_19347596	A/G	250/11	−0.40	7.47 × 10^−5^	0.06	0.36

^a^ TL, tunnel length in cm; SD, stem damage in percentage; and KR, kernel resistance on a subjective visual scale of 1 to 9 in which 1 indicates completely damaged and 9 indicates no damage.

^b^ The number before the underscore (_) indicates the chromosome number and the number after the underscore (_) indicates the physical position in bp within the chromosome.

^c^ The letter before the diagonal (/) is the nucleotide more frequent ; and the letter after the diagonal the nucleotide less frequent.

^d^ N° = number of inbred lines homozygous for a determined allelic variant. The number before the diagonal (/) represents the number of individuals with the mayor allele; and the number after the diagonal represents the number of individuals with the minor allele.

^e^ The additive effect was calculated as half the difference between the mean of the homozygous for the minor and the mean of the homozygous for the major allele.

^f^ *R* ^*2*^, proportion of the phenotypic variance explained by the SNP.

^g^ RMIP, resample model inclusion probability.

^h^ Based on SNPs from Illumina chip, the remaining locations without a superscript are based on SNPs obtained by GBS.

Nineteen SNPs were significantly associated with PH and 48 and 43 SNPs were significantly associated with DTA and DTS, respectively (Additional file 1: Table S5, S6, and S7). But none of those SNPs coincide or are close to those detected for resistance traits.

### Candidate genes selection

The filtered predicted gene set from the annotated B73 reference maize genome [60] was used to characterize the gene containing or nearby the SNP declared significant. Seven candidate genes containing or adjacent to the SNPs significantly associated with TL, four candidate genes containing or adjacent to the significant SNPs associated to SD, and ten candidate genes containing or adjacent to the significant SNPs associated to KR were proposed (Table 4).

**Table 4:** **Candidate genes for each significantly SNP associated with TL, SD, and KR and its respective encoding product**

**Chr** ^**a**^	**Trait**	**SNP physical position (bp)**	**Gene ID**	**Encoding**
2	TL	168,004,182	GRMZM2G504910	Tetratricopeptide repeat (TPR)
3	TL	7,081,859	GRMZM2G104081^b^	*hex1* (*hexokinase1*)
4	TL	190,444,179	GRMZM2G013128^b^	Double Clp-N motif-containing P-loop nucleoside triphosphate hydrolases superfamily protein
4	TL	190,679,094	GRMZM2G033820	Phospholipase A2
7	TL	154,739,818	GRMZM2G077008^**bc**^	Histidine kinase, hybrid-type, ethylene sensor
		154,741,622		
7	TL	155,702,328	GRMZM2G861541	Expressed protein
10	TL	133,337,924	GRMZM2G057084	Calcium/calmodulin-dependent protein kinase
		133,337,925		
		133,337,950		
1	SD	208,315,891	GRMZM2G101422	Expressed protein
1	SD	293,163,491	GRMZM2G060702^b^	Actin depolymerizing factor 4
2	SD	59,729,532	GRMZM2G389097	Leucine-rich receptor-like protein kinase family
5	SD	176,870,721	GRMZM2G325683^**c**^	Expressed protein
3	KR	187,742,562	GRMZM2G438551	*cystatin1*
3	KR	204,458,505	GRMZM2G111666	basic Helix-Loop-Helix (bHLH) transcription f.
3	KR	204,586,960	GRMZM2G091494^c^	Starch branching enzyme interacting protein-1
3	KR	222,733,400	GRMZM2G055578^c^	Glycine-rich protein
			GRMZM2G055629	Plant thionin family protein precursor/Defensin
4	KR	227,101,950	GRMZM2G116314	Ubiquitin thiolesterase
		227,101,985		
5	KR	93,580,059	GRMZM2G037308^c^	Phytosulfokine receptor
6	KR	88,149,024	GRMZM5G876960	Polyamine oxidase (propa-1,3-diamine-forming)
		88,149,036		
7	KR	15,072,370	GRMZM2G316256	Catalase//L-ascorbate peroxidase
7	KR	19,347,596	GRMZM2G042627	Kinase associated protein phosphatase

^a^ Chr, chromosome; TL, tunnel length; SD, stem damage; and KR, kernel resistance.

^b^ Gene containing the significant SNP within an exonic region.

^c^ Gene containing the significant SNP within an intronic region.

**Figure 2:**
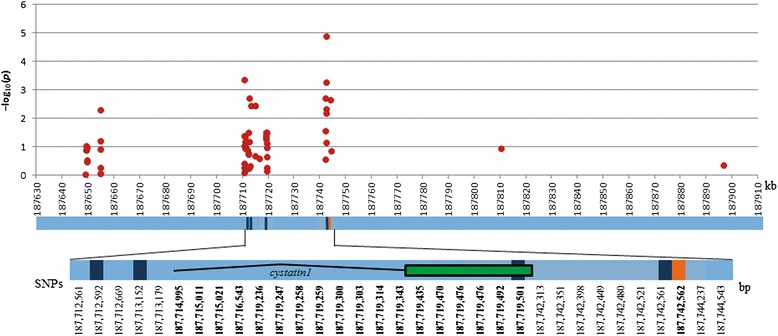
**Linkage disequilibrium heat chart showing LD measure (** ***r*** ^**2**^ **) between the SNP significantly associated with traits related to resistance to MCB attack and the closest 60 SNPs.** Each bar represent a region (ranging from ~0.15 to ~1 Mbp) containing each significant SNP associated (black square) to resistance traits. The LD (value of *r* ^*2*^) with the 30 upstream SNPs were shown at the right side of the black square, and the LD with the 30 downstream SNPs were shown at the left side of the black square; on each bar, the extreme distances (in kbp) covered by the upstream and downstream SNPs are indicated.

In general, the LD (*r*^*2*^) between significantly associated SNPs and SNPs around them decays (*r*^*2*^ ≤ 0.10) rapidly; but, in some cases, the LD spans as much as 0.5 Mb at an *r*^*2*^ value greater than 0.20 (Figure 2). For each trait, LD estimates between significant SNPs were always below 0.1 except for SNPs significant for TL located on chromosome 7 as shown in Additional file 1: Table S8.

## Discussion

### Means, heritabilities, and correlations

Substantial differences among yearly means for TL made by MCB were observed across environments, with the highest values of TL observed in 2012. Artificial infestation is used to ensure the contact of the insect with the plant, but the severity of the attack is conditioned by environmental factors that in turn influence natural infestation. The prevalence of the pest can vary greatly between and within locations [5,6]. Data on the monthly samplings at different locations in Pontevedra (unpublished data) indicate a higher rate of natural infestation in 2012 compared with 2010 and 2011 in a plot adjacent to the GWAS trial. In addition, the experiment was harvested earlier in 2011 compared with 2010 and 2012, limiting the time that larvae could damage the plants. Therefore, differences on infestation levels and harvest time could be major causes of the observed differences in TL among years.

Significant genotype × environment interaction was observed for all traits, although no G × E interaction for TL was found in previous studies under infestation with MCB [18,61]. However, G × E interaction for resistance traits was not significant, except for KR, when we made the analysis discarding data from 2012. Therefore, the high values for TL in 2012 could be obscuring the genotype effect. In previous studies, typically no G × E interaction for KR was found [61-63], with some exceptions [18].

The heritabilities for resistance traits ranged from low to moderate while PH and flowering time were higher as expected. The heritability for TL estimated herein is within the range (*h*^2^ = 0.40-0.77) of those obtained in other works with biparental crosses under infestation with MCB or ECB [16,18,64].

A significant genetic relationship was observed between TL and PH (*r*_*g*_ = 0.51) in a panel of diverse origin. Nevertheless, we did not find a single SNP or linked SNPs associated with both traits, suggesting that it is primarily due to the polygenic background. This relationship has also been important in some previous experiments with MCB and ECB [16,32], but negligible in others [17,30].

A high genetic correlation coefficients between KR and flowering time (*r*_*g*_ = 0.75 for DTA and 0.71 for DTS) and between KR and PH were found, which suggest that late and taller genotypes will be the healthiest; but these results have to be taken with caution because infestation was made simultaneously for all genotypes placing the MCB eggs close to the ground and the higher distance between the eggs and the ear of the taller and later plants could have impeded the larval arrival to the ear. In addition, when stem tissue is more lignified at time of infestation (as would be the case for earlier flowering genotypes), the preference of MCB larvae for the stem tissue compared to ear tissue could be less evident.

### Association analysis

There was a minimal variation in model fit of the compressed MLM among different traits because similar compression level values were observed for all traits (*c* = 1.1 to 1.6), except for TL, which had the highest value for compression level (*c* = 8.3). However, Zhang et al. [65] shown that this method controls the false positive rate well when the compression levels ranged from 1.5 to 10.

SNPs located on chromosomes 3 and 7 for TL co-localized with previously reported QTLs for TL by corn borers in genome bins 3.02 and 7.03 [30]. The proportion of the phenotypic variance explained (*R*^2^ = 7 - 9%) by each SNP was comparable to that explained by QTLs for TL made by ECB and MCB (*R*^2^ = 3.5 – 15.7%) in biparental crosses [16-18,64]. No QTLs for SD and KR have been previously reported in biparental crosses in the same regions where we located the significant SNPs, except one QTL for KR made by MCB at the bin 5.04 [66]. Therefore, association mapping uncovers additional genomic regions involved in maize resistance to corn borers that were not detected using biparental populations.

### Candidate genes

We used the maize B73 genome v2 (RefGen_v2) available from the Maize GDB [67] (http://www.maizegdb.org/) to identify genes that either include or are close to the significantly associated SNPs. A region of approximately 0.2 Mb around the SNP was checked for annotated genes putatively involved in plant response to wounding and/or damage by microbes (insects or pathogens) based on bibliographic records.

### Genes associated with TL made by MCB

The candidate gene adjacent to the significant SNP associated to TL on chromosome 2 encodes a Tetratricopeptide repeat (TPR) protein containing. The TPR is one of many repeat motifs that form structural domain mediating protein-protein interactions in several cellular process including translocation and degradations of proteins [68,69]. A recent study has proposed that the presence of those protein-protein interaction motifs could be acting as a modulator of the gene function and protein expression during the stress response caused by invading pathogens [70]. The *hex1* gene containing the significant SNP on chromosome 3 encodes *hexokinase*, a sugar sensor with numerous physiological functions within the cell including response to oxidative-stress and pathogen resistance [71-73]. A gene located on chromosome 4 that encodes a Double Clp-N motif-containing P-loop nucleoside triphosphate hydrolase superfamily protein contains a significant SNP. This gene shows a weak similarity to the AtHSP101 gen in *Arabidopsis*, that codifies for a heat shock protein required for acclimation to high temperatures, and probably could be involved in response to other stresses [74]. Furthermore the *ofp44* (OVATE-transcription factor 22) gene is relatively close to this SNP. It is known that some transcription factors from the Ovate family protein interact with other transcription factor families such as NAC domain protein1, MYB transcription factors, and KNOX homeodomain protein to regulate the synthesis of the three major components of secondary cell wall (lignin, cellulose and hemicellulose) in *A. thaliana* [75-78] and plants with a fortified cell wall are more resistance to corn borer attack [79].

The candidate gene for the other significant SNP on chromosome 4 encodes a Phospholipase A2 (PLA_2_) which plays a very important role in signal transduction in plants since it is the precursor of oxylipins and jasmonated acid, two hormones which regulates defense genes against herbivores [80-82].

A 1 Mbp region on chromosome 7 contains three SNPs that were significantly associated with TL and were in significant LD with at least one of the other associated SNPs in the region (*r*^2^ > 0.2; Additional file 1: Table S8). The SNPs at 154,739,818 and 154,741,622 bp are both located within a gene that putatively encodes for a Histidine kinase, hybrid-type, ethylene sensor; five other genes encoding Serine/Threonine kinase receptor and receptor-like ser/thre kinases family proteins (RLK) were also physically nearby. It is well known that both kinases and RLK proteins play a central role in signaling during pathogen recognition and the subsequent activation of plant defense mechanisms [83-85]. They are also involved in wound-mediated defense response [86], and maintenance of plant cell wall integrity [87]. Polymorphisms at the proposed kinase genes could also be responsible for the QTLs at bin 7.03 detected for TL in a biparental population [30]. The candidate gene encoding the maize Calcium/calmodulin-dependent protein kinase (CDPK) close to the three significant SNPs on chromosome 10, could be playing an important role in the activation of defense against the attack of MCB since it has been known that the CDPK is induced by mechanical wounding by herbivore attack inducing accumulation of jasmonic acid in maize and other species [88-91].

### Genes associated with SD made by MCB

A gene encoding an Actin polymerizing factor 4 (APF4) contains the second SNP at chromosome 1 significantly associated to SD. One of the functions of APF4 protein is remodeling the actin of cytoskeleton under different stimulus, including wounding and pathogen attacks [92]. Some studies in *Arabidopsis* indicated that the APF4 mediated defense signal and it is also relates with actin dynamic of cytoskeleton during the innate immune signaling [93-95]. The candidate gene for the SNP significantly associated to SD on chromosome 2 encodes a Leucine-rich receptor-like protein kinase (LRR-RLK). LRR-RLK protein family plays an important role in cell-cell signaling and other signals involving peptide in ligands. They are involved in systemic activation of protease inhibitors in response to wounding by insect feeding [96,97]. On the other hand, the significant SNP located on chromosome 5 is within a gene encoding a protein with unknown function, and it is interesting that the SNP is close to the gene *nactf30* which encodes a NAC domain protein transcription factor. As already mentioned, this gene and other transcription factor family members regulate the synthesis of secondary cell wall [98,99]. In addition, other authors have associated the NAC domain proteins with response to stresses made by herbivore attack [100].

### Genes associated with KR made by MCB

**Figure 3:**
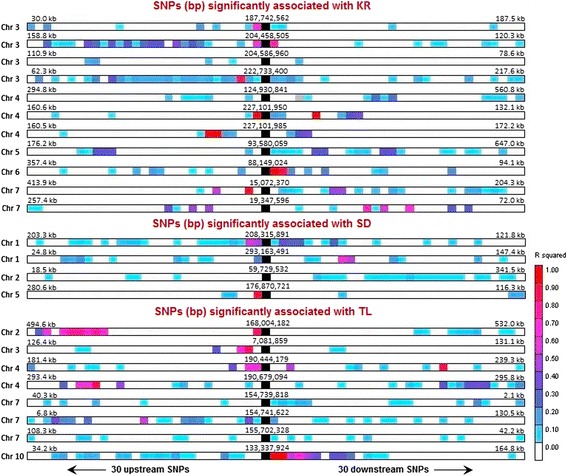
**A region of approximately 280 kpb in chromosome 3 where a SNP significantly associated with borer kernel resistance (KR) has been found.** The red points represent the *P*-values of the SNPs in the region mentioned above. The orange strip indicates the location of the SNP significantly associated with KR and the dark blue strips indicate genomic locations in LD (*r* ^*2*^ > 0.2) with the SNP significantly associated with KR. The solid black line and the green rectangle indicate the intronic and exonic region of the *cystatin1* gene, respectively. The SNP significantly associated with KR herein is in LD with the exonic region of the *cystatin1* gene, which encodes a maize cysteine proteinase inhibitor involved in plant resistance to insects.

The SNP on chromosome 3 significantly associated to KR was located nearby the c*ystatin1* gene, whose product is the corn kernel cysteine proteinase inhibitor//cysteine proteinase inhibitor I (*psei1*), an anti-metabolic protein synthetized and stored in the maize kernel [101]. The expression of some proteinase inhibitor genes are induced in response to mechanical wounding and insect damage [102], and it has been shown that cysteine proteinase inhibitor interferes with the digestive process of insects [103,104]. LD is low in this region, but it is interesting that the SNP significantly associated with KR was in LD with a SNP positioned in the exonic region of the gene (Figure 3). Therefore, if the association found between the SNP at position 187,742,562 and KR is due to the linkage between that SNP and a certain undetected polymorphism in the *cysteine1* gene, the effect of this polymorphism would be expected to be especially large because the linkage between the significant SNP and SNPs in the *cystatin1* gene is low, although significant.

Another significant SNP associated to KR was located close to a gene encoding a basic Helix-Loop-Helix (bHLH) transcription factor family member. Recent studies in other plant species have demonstrated that this family gene protein has an important role in regulating jasmonic acid response [105-109]. The third significant SNP associated with KR located on chromosome 3 was within a gene encoding a starch branching enzyme interacting protein, whose deficiency leads to decreased digestibility of maize kernel [110]. No previous evidence about its involvement in resistance to insect was found, however. A gene that encodes a Germin 1–2 protein is 106 kb downstream from this associated SNP; these proteins are implicated in the response to abiotic and biotic stresses including the response to mechanical wounding and insect damage [111-114]. Another significant SNP located on chromosome 3 at position 222,733,400 is within a gene that encodes a Glycine rich protein (GRP), a structural protein which could be playing an important role in the fortification of plant cell wall [115-117], also this protein is wound-inducible [118,119]. A set of plant Thionin family protein precursor genes were found nearby the SNP on chromosome 3 at position 222,733,400. This finding is particularly interesting since these proteins belong to the Defensin family protein which has antibacterial, antifungal, and insecticide activities [120-124].

The two significant SNPs associated to KR on chromosome 4 are adjacent to a gene that encodes for an Ubiquitin thiolesterase. These proteins form complex systems of selective protein degradation [125,126] and mediate the biosynthesis of plant hormone signaling such as salicylic, jasmonic, and abscisic acid and auxins [127,128].

The candidate gene containing the significant SNP associated to KR on chromosome 5 encodes a Phytosulfokine receptor (PSK), a recognition hormone whose level of expression has been increased by pathogens elicitors [129,130].

The candidate gene, adjacent to the significant SNP located on chromosome 6, encodes a Polyamine oxidase (propa-1,3-aimine-forming) (PAO). PAO plays an important role in stress tolerance by generating H_2_O_2_ which is a key component in signal transduction pathways leading to stress responses. In *Zea mays*, the function of PAO in wound-healing is likely due to increased lignin and suberine deposition as consequence of H_2_O_2_ release [131]. In addition, it has been described to play a role in cell wall stiffening and mediating abiotic and biotic stresses [132]. The candidate adjacent gene to the significant SNP associated to KR on chromosome 7 encodes a Catalase//L-ascorbate peroxidase, they are two major hydrogen peroxide-detoxifying enzymes whose activity is very important in the reduction of the oxidative stress caused by H_2_O_2_ [133,134], and the importance of these detoxifying enzymes in resistance to insect attack has been recently reported [135].

A candidate gene for the significant SNP on chromosome 7 at position 19,347,596 is adjacent to the SNP that encodes a Kinase associated protein phosphatase. This gene family has been proposed to regulate the response to different type of stresses including pathogens and herbivore attack [136]. It is interesting to note that another close gene to this SNP is the *ipt4* (*isopentenyl transferase4*) gene, which is involved in the regulation of cytokinin biosynthesis pathway [137]. The expression of an *itp* gen (fused with a wound-inducible promoter) in transgenic plants of *Nicotiana plumbaginifolia* decreased leaf consumption by *Manduca sexta* (lepidopterous) and reduced survival of *Myzus persicae* (aphid) [138]. Although there are several reports about the importance of cytokinins in the modulation of plant defense against pathogens and insect attack, their role is not clear [139].

Unlike markers linked to QTLs for resistance to corn borers, the SNPs associated with resistance in the present study could be incorporated in whole-genome predictor models in order to improve genomic selection [140]. Marker–assisted selection has proved an useful tool for improving resistance to the European corn borer [141] of segregating bi-parental populations related to the mapping populations used for QTL detection, but could be inappropriate in non-structured populations. As additive effects are the most important genetic effects for resistance traits, crossing inbreds with improved resistance will render hybrids more resistant to attack by MCB larvae.

## Conclusion

We conducted a genome wide association study for resistance traits to MCB with more than 245 kb SNP distributed through the whole genome. We found a set of significant SNPs associated to the three resistance traits to MCB attack. Of which 10 SNPs were significant associated to TL, 4 SNPs were associated to SD, and 11 SNPs were associated to KR. In general, each of these SNPs explain a considerable proportion of the phenotypic variance (*R*^*2*^ = 6-9%). No co-localized SNPs were found for resistance and agronomic traits that could underlie the genetic correlations found between these traits.

Twenty one candidate genes were proposed for the three resistant traits, they were either containing or adjacent (within a window of ±130 kbp) to each significantly associated SNP.

Most of the candidate genes proposed herein are part of the signaling pathway, others act as regulator of expression under biotic stress condition, and a few genes are encoding enzymes with antibiotic effect against insects such as the *cystatin1* gen and the defensin proteins.

The identification of these polymorphisms associated to resistance traits to MCB attack can be useful to understand the molecular mechanisms that affect resistance and susceptibility of host plants to insect attack, in order to contribute to advance in the understanding of plant-insect interactions. Nevertheless further studies are necessary to validate the candidate genes identified herein.

## Methods

### Plant material and phenotypic data

The maize diversity panel (composed of 302 inbred lines) represents much of the diversity available in public breeding sector around the world. A subset of the maize diversity panel (henceforth we will refer to this population as “association panel”) was evaluated for resistance to MCB attack at Pontevedra (42°24’ N, 8°38’ W, and 20 m above sea level), Spain, through three years (2010, 2011, and 2012). A subset of 270 inbred lines was assayed in an 18 × 15 α-lattice design with two replications in 2010 and 2011. In the third year a subset of 255 inbred lines was assayed (because we did not have enough seed for the remaining15 lines) in a 17 × 15 α-lattice design with two replications.

The trials were hand-planted and each experimental plot consisted of one row spaced 0.8 m apart from the other row with 29 two-kernel hills spaced 0.18 m apart. Plots were overplanted and thinned, obtaining a final density of ~70,000 plant ha^−1^. The evaluations were performed under artificial infestation with eggs of MCB. The eggs for inoculation were obtained at the Misión Biológica de Galicia by rearing the insect as described by Eizaguirre and Albajes [142]. Five plants of each plot were infested with ~ 40 MCB eggs placed between the stem and the sheath of a basal leaf.

Data collected were: tunnel length (TL), the mean length of stem tunnels made by borers on the five infested plants, which were longitudinally split at the time of harvest; stem damage (SD) as the percentage of the stem damaged by MCB larvae on the five infested plants; kernel resistance to borer attack (KR) recorded at harvest as the damage on the main ear of the five infested plants according to a subjective visual resistance scale of 1 to 9 in which 1 indicates completely damaged and 9 indicates no damage; days to anthesis (DTA) and days to silking (DTS) as the days from planting to the date on which 50% of plants were shedding pollen or showing silks, respectively; and plant height (PH) on five representative plants as the distance from the ground to the top of the plant.

### Genotypic data

We used a set of unique SNP markers derived from a Illumina maize 50 k array [56] and a genotyping-by-sequencing (GBS) strategy [48]. The two data sets were combined and filtered to exclude SNPs with more than 20% missing genotype data and minor allele frequency (MAF) less than 5%. Heterozygous genotypes were considered as missing data in the analysis. After filtering, a total of 246,477 SNPs (Additional file 2) distributed across the maize genome were used in this study.

A genetic kinship matrix (K) previously published by Olukolu et al. [52] was used for GWAS. The kinship matrix was estimated using a subset of 5000 SNPs without any missing genotypes and distributed approximately uniformly across the entire genome (at least 60 kbp between any two markers).

### Statistical analyses

#### Best linear unbiased estimator (BLUE)

Each trial was analyzed separately with the SAS mixed model procedure (PROC MIXED) in SAS software version 9.3 [143] considering inbred lines as a fixed effect and replications and block within replication as random effects. Then, trials were combined using a mixed linear model across years and considering inbred lines as the only fixed effect. As large predicted values for stem damage and tunnel length were associated with larger residuals, a natural logarithmic transformation of TL and SD scores was used for obtaining BLUEs. The logarithmic transformation eliminated the relationship between residual variance and predicted values. Line BLUEs were back-transformed and then used to perform GWAS.

#### Heritabilities

Heritabilities (*ĥ*^2^) for each year were estimated for each trait on a family-mean basis as described previously by Holland et al. [144]. The model for these analyses was similar to the model mentioned above with the exception that inbred lines were treated as random effects. The genetic (*r*_*g*_) and phenotypic (*r*_*p*_) correlations between traits were computed using REML estimation in SAS mixed model procedure following Holland [145].

### Association analysis

Genome-wide association analysis based on mixed linear model (MLM) was performed in Tassel 4.1.26 [146]. The MLM used by Tassel was

$$ \mathrm{y}=\mathrm{X}\beta +\mathrm{Z}\mathrm{u}+\mathrm{e} $$

where **y** is the vector of phenotypes (BLUEs), **β** is a vector of fixed effects, including the SNP marker tested, **u** is a vector of random additive effects (inbred lines), **X** and **Z** represents matrices, and e is a vector of random residuals. The variance of random line effects was modeled as $\mathrm{V}\mathrm{a}\mathrm{r}\left(\mathrm{u}\right)=\mathbf{K}{\sigma}_a^2$, where **K** is the *n* × *n* matrix of pairwise kinship coefficient and ${\sigma}_a^2$ is the estimated additive genetic variance [147].

Restricted maximum likelihood estimates of variance components were obtained by using the optimum compression level (compressed MLM) and population parameters previously determined options (P3D) in Tassel [65]. The optimum compression level option reduces the computation demand by clustering the (*n*) total individuals into (*s*) groups based on their realized genomic relationships, allowing the original **K** matrix to be replaced by a smaller relationship matrix. The P3D option uses iteration to estimate population parameters such as genetic and residual variance only once in a model with no fixed marker effects, then uses those estimates without iteration in subsequent association tests for each marker. The combination of these two methods reduces computational time and improves model fit [65].

### Threshold for GWAS

To identify SNPs with the most robust associations with traits, a subsampling or subagging procedure was employed in GWAS analysis [148,149]. Each of 100 subsampled datasets generated using the R software [150] comprised a random sample of 80% of inbred lines from the diversity population. Only SNP markers determined as significant at *P* < 1 × 10^−4^ and subsequently detected in ≥ 30 subsamples, i.e. resample model inclusion probability (RMIP) threshold of 0.30, were considered as significantly associated to the trait under study.

### Linkage disequilibrium and candidate gene selection

We examined the linkage disequilibrium (LD) measure (*r*^2^) with each SNP significantly associated with resistance traits in a region of 60 SNPs (ranging from 0.15 to 1 Mbp). For each trait, the linkage disequilibrium between significant SNPs was also calculated. The genes containing or adjacent to SNPs significantly associated with traits were identified and characterized by the use of MaizeGDB genome browser [67]. We examined a region around each significant SNP in order to identify candidate genes of interest. For most SNPs, more than two genes that could be involved in plant defense mechanism (based on previously published evidence) were preselected; the closest gene to the SNPs significantly associated with resistance was selected as candidate gene and the remaining genes were presented as other interesting genes that could be putatively involved in plant defense against insect herbivores.

## Additional files

Additional file 1:
**Table S1.** Mean squares and heritability estimates for MCB pest resistance traits evaluated in an association panel in three years. **Table S2** Mean squares and heritability estimates for agronomic traits evaluated in an association panel in three years. **Table S3** Phenotypic data of three resistance traits to MCB attack and three agronomic traits in a maize diversity panel. **Table S4** Annual and average means for traits related to resistance to MCB and three agronomic traits. **Table S5** Summary of the SNPs significantly associated to plant height. SNP identification (SNP ID), additive effect and allelic variants for the SNP, proportion of total variance explained by the SNPs significantly associated with plant height (PH) and significance values for the association between the SNP and the phenotype (*P*-value and RMIP). **Table S6** Summary of the SNPs significantly associated to days to anthesis. SNP identification (SNP ID), additive effect and allelic variants for the SNP, proportion of total variance explained by the SNPs significantly associated with days to anthesis (DTA) and significance values for the association between the SNP and the phenotype (*P*-value and RMIP). **Table S7** Summary of the SNPs significantly associated to days to silking. SNP identification (SNP ID), additive effect and allelic variants for the SNP, proportion of total variance explained by the SNPs significantly associated with days to silking (DTS) and significance values for the association between the SNP and the phenotype (*P*-value and RMIP). **Table S8** Linked disequilibrium (*r* ^*2*^) between the ten SNPs significantly associated to TL made by MCB. Additional file 2:
**Genotypic data of a maize diversity panel with more than 245 kb SNP (GBS + Illumina).** The data were filtered to exclude SNPs with more than 20% missing genotype data and minor allele frequency (MAF) less than 5%. Also the heterozygous are excluded. The array has 290 columns and 246,477 rows.

## Abbreviations

APF4: Actin polymerizing factor 4
bHLH: Basic Helix-Loop-Helix
BLUE: Best linear unbiased estimator
Bt: Bacillus thuringiensis
CDPK: Calcium/calmodulin-dependent protein kinase
DTA: Days to anthesis
DTS: Days to silking
ECB: European corn borer
GBS: Genotyping by sequencing
GRP: Glycine rich protein
GWAS: Genome-wide association study
KR: Kernel resistance
LD: Linkage disequilibrium
LRR-RLK: Leucine-rich receptor-like protein kinase
MAF: Minor allele frequency
MCM: Mediterranean corn borer
PAO: Polyamine oxidase
PH: Plant height
PLA2: Phospholipase A2
PSK: Phytosulfokine receptor
QTL: Quantitative trait locus/loci
REML: Restricted maximum likelihood
RLK: Receptor like kinase
RMIP: Resample model inclusion probability
SD: Stem damage
SNP: Single nucleotide polymorphism
TL: Tunnel length
TPR: Tetratricopeptide repeat

## References

[1] Meihls LN, Kaur H, Jander G (2012). Natural variation in maize defense against insect herbivores. Cold Spring Harb Sym.

[2] Malvar RA, Cartea ME, Revilla P, Ordás A, Alvarez A, Mansilla JP (1993). Sources of resistance to pink stem borer and European corn borer in maize. Maydica.

[3] Avantaggiato G, Quaranta F, Desiderio E, Visconti A (2002). Fumonisin contamination of maize hybrids visibly damaged by *Sesamia*. J Sci Food Agric.

[4] Visconti A, Marasas WFO, Miller JD, Riley R (1999). Mycotoxins of growing interest: Fumonisins. Book Mycotoxins of growing interest: Fumonisins.

[5] Cordero A, Malvar RA, Butrón A, Revilla P, Velasco P, Ordás A (1998). Population dynamics and life-cycle of corn borers in South Atlantic European Coast. Maydica.

[6] Velasco P, Revilla P, Monetti L, Butrón A, Ordás A, Malvar RA (2007). Corn borers (Lepidoptera: Noctuidae; Crambidae) northwestern Spain: population dinamics and distribution. Maydica.

[7] Meissle M, Romeis J, Bigler F (2011). *Bt* maize and integrated pest management - a European perspective. Pest Manag Sci.

[8] Speiser B, Tamm L, Ehlers R-U (2011). Regulation of plant protection in organic farming. Regulation of Biological Control Agents.

[9] Campagne P, Kruger M, Pasquet R, Le Ru B, Van den Berg J (2013). Dominant inheritance of field-evolved resistance to *Bt* corn in *Busseola fusca*. PLoS One.

[10] Tabashnik BE, Brévault T, Carrière Y (2013). Insect resistance to Bt crops: lessons from the first billion acres. Nat Biotechnol.

[11] González-Cabrera J, García M, Hernández-Crespo P, Farinós GP, Ortego F, Castañera P (2013). Resistance to Bt maize in *Mythimna unipuncta* (Lepidoptera: Noctuidae) is mediated by alteration in Cry1Ab protein activation. Insect Biochem Mol Biol.

[12] Gould F (1998). Sustainability of transgenic insecticidal cultivars: integrating pest genetics and ecology. Annu Rev Entomol.

[13] Butrón A, Malvar R, Velasco P, Vales M, Ordás A (1999). Combining abilities for maize stem antibiosis, yield loss, and yield under infestation and non infestation with pink stem borer. Crop Sci.

[14] Cartea ME, Malvar RA, Butrón A, Vales MI, Ordás A (1999). Inheritance of antibiosis to *Sesamia nonagrioides* (Lepidoptera: Noctuidae) in maize. J Econ Entomol.

[15] Papst C, Bohn M, Utz HF, Melchinger AE, Klein D, Eder J (2004). QTL mapping for European corn borer resistance (*Ostrinia nubilalis* Hb.), agronomic and forage quality traits of testcross progenies in early- maturing European maize (*Zea mays* L.) germplasm. Theor Appl Genet.

[16] Schön CC, Lee M, Melchinger AE, Guthrie WD, Woodman WL (1993). Mapping and characterization of quantitative trait loci affecting resistancie against second-generation European corn borer in maize with the aid of RFLPs. Heredity.

[17] Cardinal AJ, Lee M, Sharopova N, Woodman WL, Long MJ (2001). Genetic mapping and analysis of quantitative trait loci for resistance to stalk tunneling by European corn borer in maize. Crop Sci.

[18] Ordás B, Malvar RA, Santiago R, Sandoya G, Romay MC, Butrón A (2009). Mapping of QTL for resistance to the Mediterranean corn borer attack using the intermated B73 × Mo17 (IBM) population in maize. Theor Appl Genet.

[19] Klenke J, Russel W, Guthrie W (1986). Recurrent selection for resistance to European corn borer in a corn synthetic and correlated effects on agronomic traits. Crop Sci.

[20] Bosque-Pérez N, Kling J, Odubiyi S (1997). Recent advances in the development of sources of resistance to pink stalk borer and African sugarcane borer. Insect resistant maize: Recent advances and utilization Proc of an International Symposium.

[21] Sandoya G, Butrón A, Alvarez A, Ordás A, Malvar RA (2008). Direct response of a maize synthetic to recurrent selection for resistance to stem borers. Crop Sci.

[22] Butrón A, Romay MC, Peña-Asin J, Alvarez A, Malvar RA (2012). Genetic relationship between maize resistance to corn borer attack and yield. Crop Sci.

[23] Ordás B, Butrón A, Alvarez A, Revilla P, Malvar R (2012). Comparison of two methods of reciprocal recurrent selection in maize (*Zea mays* L.). Theor Appl Genet.

[24] Malvar RA, Butrón A, Revilla P, Ordás A (2004). Resistance to the pink stem borer, *Sesamia nonagrioides*, in maize. Recent Res Devel Plant Sci.

[25] Cartea ME, Malvar RA, Vales I, Butrón A, Ordás A (2001). Inheritance of resistance to ear damage caused by *Sesamia nonagrioides* (Lepidoptera: Noctuidae) in maize. J Econ Entomol.

[26] Hudon M, Chiang MS (1991). Evaluation of resistance of maize germplasm to the univoltine European corn borer *Ostrinia nubilalis* (Hübner) and relationship with maize maturity in Quebec. Maydica.

[27] Guthrie W, Russell W (1989). Breeding methodologies and genetic basis of resistance in maize to the European corn borer. International Symposium on Methodologies for Developing Host Plant Resistance to Maize Insects Mexico, DF (Mexico) 9–14 Mar 1987.

[28] Krakowsky MD, Brinkman MJ, Woodman WL, Lee M (2002). Genetic components of resistance to stalk tunneling by the European corn borer in maize. Crop Sci.

[29] Krakowsky MD, Lee M, Holland JB (2007). Genotypic correlation and multivariate QTL analyses for cell wall components and resistance to stalk tunneling by the European corn borer in maize. Crop Sci.

[30] Krakowsky MD, Lee M, Woodman WL, Long MJ, Sharopova N (2004). QTL mapping of resistance to stalk tunneling by the European corn borer in RILs of maize population B73 × De811. Crop Sci.

[31] Orsini E, Krchov L, Uphaus J, Melchinger A (2012). Mapping of QTL for resistance to first and second generation of European corn borer using an integrated SNP and SSR linkage map. Euphytica.

[32] Ordás B, Malvar RA, Santiago R, Butrón A (2010). QTL mapping for Mediterranean corn borer resistance in European flint germplasm using recombinant inbred lines. BMC Genomics.

[33] Bohn M, Khairallah MM, González-de-León D, Hoisington DA, Utz HF, Deutsch JA (1996). QTL mapping in tropical maize: I. Genomic regions affecting leaf feeding resistance to sugarcane borer and other traits. Crop Sci.

[34] Groh S, González-de-León D, Khairallah MM, Jiang C, Bergvinson D, Bohn M (1998). QTL mapping in tropical maize III. Genomic regions for resistance to Diatraea spp and associated traits in two RIL populations. Crop Sci.

[35] Bohn M, Khairallah M, Jiang C, González-de-León D, Hoisington D, Utz H (1997). QTL mapping in tropical maize: II. Comparison of genomic regions for resistance to *Diatraea spp*. Crop Sci.

[36] Weiss LA, Arking DE, Daly MJ, Chakravarti A, Brune CW, West K (2009). A genome-wide linkage and association scan reveals novel loci for autism. Nature.

[37] Duerr RH, Taylor KD, Brant SR, Rioux JD, Silverberg MS, Daly MJ (2006). A genome-wide association study identifies IL23R as an inflammatory bowel disease gene. Science.

[38] Sladek R, Rocheleau G, Rung J, Dina C, Shen L, Serre D (2007). A genome-wide association study identifies novel risk loci for type 2 diabetes. Nature.

[39] Barendse W, Reverter A, Bunch RJ, Harrison BE, Barris W, Thomas MB (2007). A validated whole-genome association study of efficient food conversion in cattle. Genetics.

[40] Kijas JW, Townley D, Dalrymple BP, Heaton MP, Maddox JF, McGrath A (2009). A genome wide survey of SNP variation reveals the genetic structure of sheep breeds. PLoS One.

[41] Bolormaa S, Hayes B, Savin K, Hawken R, Barendse W, Arthur P (2011). Genome-wide association studies for feedlot and growth traits in cattle. J Anim Sci.

[42] Fan B, Onteru SK, Du Z-Q, Garrick DJ, Stalder KJ, Rothschild MF (2011). Genome-wide association study identifies loci for body composition and structural soundness traits in pigs. PLoS One.

[43] Tian F, Bradbury PJ, Brown PJ, Hung H, Sun Q, Flint-Garcia S (2011). Genome-wide association study of leaf architecture in the maize nested association mapping population. Nat Genet.

[44] Neumann K, Kobiljski B, Denčić S, Varshney R, Börner A (2011). Genome-wide association mapping: a case study in bread wheat (*Triticum aestivum* L.). Mol Breed.

[45] Zhu C, Gore M, Buckler ES, Yu J (2008). Status and prospects of association mapping in plants. Plant Genome.

[46] Peiffer JA, Flint-Garcia SA, De Leon N, McMullen MD, Kaeppler SM, Buckler ES (2013). The genetic architecture of maize stalk strength. PLoS One.

[47] Oraguzie NC, Rikkerink EH, Gardiner SE, Silva H (2007). Association mapping in plants.

[48] Romay MC, Millard MJ, Glaubitz JC, Peiffer J, Swarts K, Casstevens TM (2013). Comprehensive genotyping of the USA national maize inbred seed bank. Genome Biol.

[49] Flint-Garcia SA, Thuillet A-C, Yu J, Pressoir G, Romero SM, Mitchell SE (2005). Maize association population: a high-resolution platform for quantitative trait locus dissection. Plant J.

[50] Liu K, Goodman M, Muse S, Smith JS, Buckler ES, Doebley J (2003). Genetic structure and diversity among maize inbred lines as inferred from DNA microsatellites. Genetics.

[51] Cook JP, McMullen MD, Holland JB, Tian F, Bradbury P, Ross-Ibarra J (2012). Genetic architecture of maize kernel composition in the nested association mapping and inbred association panels. Plant Physiol.

[52] Olukolu BA, Negeri A, Dhawan R, Venkata B, Sharma P, Garg A (2013). A connected set of genes associated with programmed cell death implicated in controlling the hypersensitive response in maize. Genetics.

[53] Zila CT, Samayoa LF, Santiago R, Butrón A, Holland JB (2013). A genome-wide association study reveals genes associated with fusarium ear rot resistance in a maize core diversity panel. G3-Genes Genomes Genet.

[54] Atwell S, Huang YS, Vilhjálmsson BJ, Willems G, Horton M, Li Y (2010). Genome-wide association study of 107 phenotypes in *Arabidopsis thaliana* inbred lines. Nature.

[55] Chan EK, Rowe HC, Kliebenstein DJ (2010). Understanding the evolution of defense metabolites in *Arabidopsis thaliana* using genome-wide association mapping. Genetics.

[56] Ganal MW, Durstewitz G, Polley A, Bérard A, Buckler ES, Charcosset A (2011). A large maize (*Zea mays* L.) SNP genotyping array: development and germplasm genotyping, and genetic mapping to compare with the B73 reference genome. PLoS One.

[57] Elshire RJ, Glaubitz JC, Sun Q, Poland JA, Kawamoto K, Buckler ES (2011). A robust, simple genotyping-by-sequencing (GBS) approach for high diversity species. PLoS One.

[58] Glaubitz JC, Casstevens TM, Lu F, Harriman J, Elshire RJ, Sun Q (2014). TASSEL-GBS: A High Capacity Genotyping by Sequencing Analysis Pipeline. PLoS One.

[59] Steel RG, Torrie JH (1960). Principles and procedures of statistics.

[60] Schnable PS, Ware D, Fulton RS, Stein JC, Wei F, Pasternak S (2009). The B73 maize genome: complexity, diversity, and dynamics. Science.

[61] Butrón A, Malvar RA, Velasco P, Cartea ME, Ordás A (1998). Combining abilities and reciprocal effects for maize ear resistance to pink stem borer. Maydica.

[62] Velasco P, Revilla P, Butrón A, Ordás B, Ordás A, Malvar RA (2002). Ear damage of sweet corn inbreds and their hybrids under multiple corn borer infestation. Crop Sci.

[63] Velasco P, Malvar RA, Butrón A, Revilla P, Ordás A (1999). Ear feeding resistance of sweet corn inbreds to pink stem borer. J Am Soc Hortic Sci.

[64] Bohn M, Schulz B, Kreps R, Klein D, Melchinger AE (2000). QTL mapping for resistance against the European corn borer (*Ostrinia nubilalis* H.) in early maturing European dent germplasm. Theor Appl Genet.

[65] Zhang Z, Ersoz E, Lai C-Q, Todhunter RJ, Tiwari HK, Gore MA (2010). Mixed linear model approach adapted for genome-wide association studies. Nat Genet.

[66] Samayoa LF, Butrón A, Malvar RA (2014). QTL mapping for maize resistance and yield under infestation with *Sesamia nonagrioides*. Mol Breeding.

[67] Harper LC, Schaeffer ML, Thistle J, Gardiner J, Andorf C, Campbell D (2011). The MaizeGDB Genome Browser tutorial: one example of database outreach to biologists via video. Database.

[68] Allan RK, Ratajczak T (2011). Versatile TPR domains accommodate different modes of target protein recognition and function. Cell Stress Chaperones.

[69] Blatch GL, Lässle M (1999). The tetratricopeptide repeat: a structural motif mediating protein-protein interactions. Bioessays.

[70] Pogorelko GV, Mokryakova M, Fursova OV, Abdeeva I, Piruzian ES, Bruskin SA (2014). Characterization of three Arabidopsis thaliana immunophilin genes involved in the plant defense response against P. syringae. Gene.

[71] Hellmann HA, Smeekens S (2014). Sugar sensing and signaling in plants. Front Plant Sci.

[72] Granot D, David-Schwartz R, Kelly G (2013). Hexose kinases and their role in sugar-sensing and plant development. Front Plant Sci.

[73] Morkunas I, Ratajczak L (2014). The role of sugar signaling in plant defense responses against fungal pathogens. Acta Physiol Plant.

[74] Lee U, Rioflorido I, Hong SW, Larkindale J, Waters ER, Vierling E (2007). The Arabidopsis ClpB/Hsp100 family of proteins: chaperones for stress and chloroplast development. Plant J.

[75] Zhong R, Richardson EA, Ye Z-H (2007). The MYB46 transcription factor is a direct target of SND1 and regulates secondary wall biosynthesis in *Arabidopsis*. Plant Cell.

[76] Zhong R, Lee C, Zhou J, McCarthy RL, Ye Z-H (2008). A battery of transcription factors involved in the regulation of secondary cell wall biosynthesis in *Arabidopsis*. Plant Cell.

[77] Liu Y (2010). Investigation of a KNAT7-BLH-OFP transcription factor complex involved in regulation of secondary cell wall biosynthesis in *Arabidopsis thaliana*. MSc Thesis.

[78] Mitsuda N, Seki M, Shinozaki K, Ohme-Takagi M (2005). The NAC transcription factors NST1 and NST2 of *Arabidopsis* regulate secondary wall thickenings and are required for anther dehiscence. Plant Cell.

[79] Barros-Rios J, Malvar RA, Jung H-JG, Santiago R (2011). Cell wall composition as a maize defense mechanism against corn borers. Phytochemistry.

[80] Ryan CA (2000). The systemin signaling pathway: differential activation of plant defensive genes. BBA-Protein Struct M.

[81] Meijer HJ, Munnik T (2003). Phospholipid-based signaling in plants. Annu Rev Plant Biol.

[82] Zhu-Salman K, Bi J-L, Liu T-X (2005). Molecular strategies of plant defense and insect counter-defense. Insect Sci.

[83] Afzal AJ, Wood AJ, Lightfoot DA (2008). Plant receptor-like serine threonine kinases: Roles in signaling and plant defense. Mol Plant-Microbe Interact.

[84] Nürnberger T, Kemmerling B (2006). Receptor protein kinases–pattern recognition receptors in plant immunity. Trends Plant Sci.

[85] Duan Y, Ge C, Liu S, Wang J, Zhou M (2013). A two-component histidine kinase *Shk1* controls stress response, sclerotial formation and fungicide resistance in *Sclerotinia sclerotiorum*. Mol Plant Pathol.

[86] G-i A, Kost C, Boland W (2005). Herbivore-induced, indirect plant defences. BBA-Mol Cell Biol L.

[87] Engelsdorf T, Hamann T (2014). An update on receptor-like kinase involvement in the maintenance of plant cell wall integrity. Ann Bot.

[88] Szczegielniak J, Borkiewicz L, Szurmak B, Lewandowska-Gnatowska E, Statkiewicz M, Klimecka M (2012). Maize calcium-dependent protein kinase (ZmCPK11): local and systemic response to wounding, regulation by touch and components of jasmonate signaling. Physiol Plant.

[89] Yang D-H, Hettenhausen C, Baldwin IT, Wu J (2012). Silencing *Nicotiana attenuata* calcium-dependent protein kinases, *CDPK4* and *CDPK5*, strongly up-regulates wound-and herbivory-induced jasmonic acid accumulations. Plant Physiol.

[90] Szczegielniak J, Klimecka M, Liwosz A, Ciesielski A, Kaczanowski S, Dobrowolska G (2005). A wound-responsive and phospholipid-regulated maize calcium-dependent protein kinase. Plant Physiol.

[91] Ma F, Lu R, Liu H, Shi B, Zhang J, Tan M (2012). Nitric oxide-activated calcium/calmodulin-dependent protein kinase regulates the abscisic acid-induced antioxidant defence in maize. J Exp Bot.

[92] Staiger CJ, Gibbon BC, Kovar DR, Zonia LE (1997). Profilin and actin-depolymerizing factor: modulators of actin organization in plants. Trends Plant Sci.

[93] Tian M, Chaudhry F, Ruzicka DR, Meagher RB, Staiger CJ, Day B (2009). Arabidopsis actin-depolymerizing factor AtADF4 mediates defense signal transduction triggered by the *Pseudomonas syringae* effector AvrPphB. Plant Physiol.

[94] Henty-Ridilla JL, Li J, Day B, Staiger CJ (2014). Actin depolymerizing factor4 regulates actin dynamics during innate immune signaling in *Arabidopsis*. Plant Cell.

[95] Porter K, Shimono M, Tian M, Day B (2012). Arabidopsis Actin-Depolymerizing Factor-4 links pathogen perception, defense activation and transcription to cytoskeletal dynamics. PLoS Pathog.

[96] Wu Y, Zhou JM (2013). Receptor-Like Kinases in Plant Innate Immunity. J Integr Plant Biol.

[97] Jones DA, Jones J (1997). The role of leucine-rich repeat proteins in plant defences. Adv Bot Res.

[98] McCann MC, Carpita NC (2008). Designing the deconstruction of plant cell walls. Curr Opin Plant Biol.

[99] Zhong R, Demura T, Ye Z-H (2006). SND1, a NAC domain transcription factor, is a key regulator of secondary wall synthesis in fibers of *Arabidopsis*. Plant Cell.

[100] Nogueira FT, Schlögl PS, Camargo SR, Fernandez JH (2005). SsNAC23, a member of the NAC domain protein family, is associated with cold, herbivory and water stress in sugarcane. Plant Sci.

[101] Abe M, Abe K, Kuroda M, Arai S (1992). Corn kernel cysteine proteinase inhibitor as a novel cystatin superfamily member of plant origin. Eur J Biochem.

[102] McMullen MD, Frey M, Degenhardt J, Bennetzen JL, Hake SC (2009). Genetics and biochemistry of insect resistance in maize. *Handbook of maize: Its biology*.

[103] Ussuf K, Laxmi N, Mitra R (2001). Proteinase inhibitors: plant-derived genes of insecticidal protein for developing insect-resistant transgenic plants. Curr Sci India.

[104] Fabrick J, Behnke C, Czapla T, Bala K, Rao A, Kramer K (2002). Effects of a potato cysteine proteinase inhibitor on midgut proteolytic enzyme activity and growth of the southern corn rootworm, *Diabrotica undecimpunctata howardi* (Coleoptera: Chrysomelidae). Insect Biochem Mol Biol.

[105] Zhang H, Hedhili S, Montiel G, Zhang Y, Chatel G, Pré M (2011). The basic helix-loop-helix transcription factor CrMYC2 controls the jasmonate-responsive expression of the *ORCA* genes that regulate alkaloid biosynthesis in *Catharanthus roseus*. Plant J.

[106] Niu Y, Figueroa P (2011). Characterization of JAZ-interacting bHLH transcription factors that regulate jasmonate responses in *Arabidopsis*. J Exp Bot.

[107] Sasaki-Sekimoto Y, Jikumaru Y, Obayashi T, Saito H, Masuda S, Kamiya Y (2013). Basic Helix-Loop-Helix transcription factors JASMONATE-ASSOCIATED MYC2-LIKE1 (JAM1), JAM2, and JAM3 are negative regulators of jasmonate responses in Arabidopsis. Plant Physiol.

[108] Qi T, Huang H, Wu D, Yan J, Qi Y, Song S (2014). Arabidopsis DELLA and JAZ Proteins Bind the WD-Repeat/bHLH/MYB Complex to Modulate Gibberellin and Jasmonate Signaling Synergy. Plant Cell.

[109] Song S, Qi T, Fan M, Zhang X, Gao H, Huang H (2013). The bHLH subgroup IIId factors negatively regulate jasmonate-mediated plant defense and development. PLoS Genet.

[110] Xia H, Yandeau-Nelson M, Thompson DB, Guiltinan MJ (2011). Deficiency of maize starch-branching enzyme i results in altered starch fine structure, decreased digestibility and reduced coleoptile growth during germination. BMC Plant Biol.

[111] Dunwell JM, Gibbings JG, Mahmood T, Saqlan Naqvi S (2008). Germin and germin-like proteins: evolution, structure, and function. Crit Rev Plant Sci.

[112] Davidson RM, Reeves PA, Manosalva PM, Leach JE (2009). Germins: A diverse protein family important for crop improvement. Plant Sci.

[113] Breen J, Bellgard M (2010). Germin-like proteins (GLPs) in cereal genomes: gene clustering and dynamic roles in plant defence. Funct Integr Genomics.

[114] Armstrong JS, Abdel-Mageed H, Fokar M, Allen R, Adamczyk JJ (2013). Dietary effects of cotton tissue expressing germin like protein on beet armyworm (Lepidoptera: Noctuidae) growth, survival and pupation. Fla Entomol.

[115] Ryser U, Schorderet M, Guyot R, Keller B (2004). A new structural element containing glycine-rich proteins and rhamnogalacturonan I in the protoxylem of seed plants. J Cell Sci.

[116] Ringli C, Keller B, Ryser U (2001). Glycine-rich proteins as structural components of plant cell walls. Cell Mol Life Sci.

[117] Mangeon A, Junqueira RM, Sachetto-Martins G (2010). Functional diversity of the plant glycine-rich proteins superfamily. Plant Signal Behav.

[118] Showalter AM (1993). Structure and function of plant cell wall proteins. Plant Cell.

[119] Sturm A (1992). A wound-inducible glycine-rich protein from Daucus carota with homology to single-stranded nucleic acid-binding proteins. Plant Physiol.

[120] Bohlmann H, Broekaert W (1994). The role of thionins in plant protection. Crit Rev Plant Sci.

[121] De Coninck B, Cammue B, Thevissen K (2013). Modes of antifungal action and *in planta* functions of plant defensins and defensin-like peptides. Fungal Biol Rev.

[122] Shiau Y-S, Horng S-B, Chen C-S, Huang P-T, Lin C, Hsueh Y-C (2006). Structural analysis of the unique insecticidal activity of novel mungbean defensin VrD1 reveals possibility of homoplasy evolution between plant defensins and scorpion neurotoxins. J Mol Recognit.

[123] Bohlmann H, Apel K (1991). Thionins. Annu Rev Plant Biol.

[124] Kessler A, Baldwin IT (2002). Plant responses to insect herbivory: The emerging molecular analysis. Annu Rev Plant Biol.

[125] Hershko A, Ciechanover A (1992). The ubiquitin system for protein degradation. Annu Rev Biochem.

[126] Zeng L-R, Vega-Sánchez ME, Zhu T, Wang G-L (2006). Ubiquitination-mediated protein degradation and modification: an emerging theme in plant-microbe interactions. Cell Res.

[127] Kelley DR, Estelle M (2012). Ubiquitin-mediated control of plant hormone signaling. Plant Physiol.

[128] Santner A, Estelle M (2010). The ubiquitin-proteasome system regulates plant hormone signaling. Plant J.

[129] Igarashi D, Tsuda K, Katagiri F (2012). The peptide growth factor, phytosulfokine, attenuates pattern-triggered immunity. Plant J.

[130] Rodriguez PA (2011). Analysis of *AtPSKR1*, an LRR receptor protein kinase, and other PSK-signalling components in plant defence responses. PhD Thesis.

[131] Angelini R, Tisi A, Rea G, Chen MM, Botta M, Federico R (2008). Involvement of polyamine oxidase in wound healing. Plant Physiol.

[132] Wimalasekera R, Tebartz F, Scherer GF (2011). Polyamines, polyamine oxidases and nitric oxide in development, abiotic and biotic stresses. Plant Sci.

[133] Rizhsky L, Hallak-Herr E, Van Breusegem F, Rachmilevitch S, Barr JE, Rodermel S (2002). Double antisense plants lacking ascorbate peroxidase and catalase are less sensitive to oxidative stress than single antisense plants lacking ascorbate peroxidase or catalase. Plant J.

[134] Willekens H, Chamnongpol S, Davey M, Schraudner M, Langebartels C, Van Montagu M (1997). Catalase is a sink for H_2_O_2_ and is indispensable for stress defence in C_3_ plants. EMBO J.

[135] Kaur R, Gupta AK, Taggar GK (2014). Role of catalase, H_2_O_2_ and phenolics in resistance of pigeonpea towards *Helicoverpa armigera* (Hubner). Acta Physiol Plant.

[136] Bajsa J, Pan Z, Duke SO (2011). Serine/threonine protein phosphatases: Multi-purpose enzymes in control of defense mechanisms. Plant Signal Behav.

[137] Vyroubalová Š, Václavíková K, Turečková V, Novák O, Šmehilová M, Hluska T (2009). Characterization of new maize genes putatively involved in cytokinin metabolism and their expression during osmotic stress in relation to cytokinin levels. Plant Physiol.

[138] Smigocki A, Neal J, McCanna I, Douglass L (1993). Cytokinin-mediated insect resistance in *Nicotiana* plants transformed with the *ipt* gene. Plant Mol Biol.

[139] Giron D, Frago E, Glevarec G, Pieterse CM, Dicke M (2013). Cytokinins as key regulators in plant-microbe-insect interactions: connecting plant growth and defence. Funct Ecol.

[140] De Los CG, Gianola D, Allison DB (2010). Predicting genetic predisposition in humans: the promise of whole-genome markers. Nat Rev Genet.

[141] Flint-Garcia SA, Darrah LL, McMullen MD, Hibbard BE (2003). Phenotypic versus marker-assisted selection for stalk strength and second-generation European corn borer resistance in maize. Theor Appl Genet.

[142] Eizaguirre M, Albajes R (1992). Diapause induction in the stem corn borer *Sesamia nonagrioides* (Lepidoptera, Noctuidae). Entomol Gen.

[143] SAS Institute Inc. SAS 9.3 Guide to software updates. Cary, NC, USA, SAS Institute Inc. 2011; 314 p

[144] Holland JB, Nyquist WE, Cervantes-Martínez CT, Janick J (2003). Estimated an interpreting heritability for plant breeding: An update. Plant Breeding Reviews.

[145] Holland JB (2006). Estimating genotypic correlations and their standard errors using multivariate restricted maximum likelihood estimation with SAS Proc MIXED. Crop Sci.

[146] Bradbury PJ, Zhang Z, Kroon DE, Casstevens TM, Ramdoss Y, Buckler ES (2007). TASSEL: software for association mapping of complex traits in diverse samples. Bioinformatics.

[147] Yu J, Pressoir G, Briggs WH, Vroh I, Yamasaki I, Doebley JF (2006). A unified mixed-model method for association mapping that accounts for multiple levels of relatedness. Nat Genet.

[148] Valdar W, Holmes CC, Mott R, Flint J (2009). Mapping in structured populations by resample model averaging. Genetics.

[149] Panagiotou OA, Ioannidis JP (2012). What should the genome-wide significance threshold be? Empirical replication of borderline genetic associations. Int J Epidemiol.

[150] R Core Team (2013). R: A languange and environment for statistical computing. Book R: A languange and environment for statistical computing (Editor ed.^eds.).

