# Supplementary material for: Genome-wide association study reveals a set of genes associated with resistance to the Mediterranean corn borer (Sesamia nonagrioides L.) in a maize diversity panel
Source: BMC Plant Biol. 2015 Feb 5;15:35. doi: 10.1186/s12870-014-0403-3 (PMC4340109; doi:10.1186/s12870-014-0403-3)
Supplement: Additional file 1: — Table S1. Mean squares and heritability estimates for MCB pest resistance traits evaluated in an association panel in three years. Table S2 Mean squares and heritability estimates for agronomic traits evaluated in an association panel in three years. Table S3 Phenotypic data of three resistance traits to MCB attack and three agronomic traits in a maize diversity panel. Table S4 Annual and average means for traits related to resistance to MCB and three agronomic traits. Table S5 Summary of the SNPs significantly associated to plant height. SNP identification (SNP ID), additive effect and allelic variants for the SNP, proportion of total variance explained by the SNPs significantly associated with plant height (PH) and significance values for the association between the SNP and the phenotype (P-value and RMIP). Table S6 Summary of the SNPs significantly associated to days to anthesis. SNP identification (SNP ID), additive effect and allelic variants for the SNP, proportion of total variance explained by the SNPs significantly associated with days to anthesis (DTA) and significance values for the association between the SNP and the phenotype (P-value and RMIP). Table S7 Summary of the SNPs significantly associated to days to silking. SNP identification (SNP ID), additive effect and allelic variants for the SNP, proportion of total variance explained by the SNPs significantly associated with days to silking (DTS) and significance values for the association between the SNP and the phenotype (P-value and RMIP). Table S8 Linked disequilibrium (r 2) between the ten SNPs significantly associated to TL made by MCB. [file 12870_2014_403_MOESM1_ESM.docx]

**Genome-wide association study reveals a set of genes associated with resistance to the Mediterranean corn borer (*Sesamia nonagrioides* L.) in a maize diversity panel.**

LF Samayoa, RA Malvar, BA Olukolu, JB Holland, and A Butrón

Corresponding author: Luis Fernando Samayoa (Misión Biológica de Galicia-CSIC)

E.mail: [fsamayoa@mbg.csic.es](mailto:fsamayoa@mbg.csic.es)

**Table S1 Mean squares and heritability estimates for MCB pest resistance traits evaluated in an association panel in three years.**

| Source | df | TL | df | SD | df | KR |
| --- | --- | --- | --- | --- | --- | --- |
| Environment (E) | 2 | 110065.8** | 2 | 29682.0** | 2 | 31.0** |
| Rep (E) | 3 | 3759.9** | 3 | 1219.0** | 3 | 17.0** |
| Genotype | 270 | 380.1** | 270 | 72.0** | 267 | 2.1** |
| Genotype × E | 515 | 142.6** | 506 | 50.1** | 508 | 1.0** |
| Error | 627 | 95.3 | 612 | 36.5 | 592 | 0.8 |
| Heritabilities (*h*^2^) (%) |  | 60 ± 4.5^†^ |  | 25 ± 8.5 |  | 52 ± 5.6 |

df, degrees of freedom; TL, tunnel length; SD, stem damage; KR, kernel resistance;

* Significant at the 0.05 probability level, ** significant at the 0.01 probability level

^†^Heritabilities (*h*^2^) ± standard error, estimated on a family mean basis following Holland et al. (2003).

**Table S2 Mean squares and heritability estimates for agronomic traits evaluated in an association panel in three years.**

| Source | df | PH | df | DTA | df | DTS |
| --- | --- | --- | --- | --- | --- | --- |
| Environment (E) | 2 | 20012.2** | 2 | 27888.1** | 2 | 28088.7** |
| Rep (E) | 3 | 4664.0** | 3 | 72.0** | 3 | 76.5** |
| Genotype | 269 | 4329.9** | 269 | 697.1** | 269 | 677.1** |
| Genotype × E | 510 | 254.1* | 519 | 15.5** | 511 | 12.0** |
| Error | 639 | 217.9 | 671 | 9.6 | 658 | 6.3 |
| Heritabilities (*h*^2^) (%) |  | 94 ± 0.6 |  | 98 ± 0.2 |  | 98 ± 0.2 |

PH, plant height; DTA, days to anthesis; DTS, days to silking.

* Significant at the 0.05 probability level, ** significant at the 0.01 probability level

^†^Heritabilities (*h*^2^) ± standard error, estimated on a family mean basis following Holland et al. (2003).

**Table S3 Phenotypic data of three resistance traits to MCB attack and three agronomic traits in a maize diversity panel**

| Inbred | TL | SD | KR | PH | DTA | DTS |
| --- | --- | --- | --- | --- | --- | --- |
| 4226 | 43.9252 | 18.4957 | 7.55729 | 197.725 | 80.946 | 82.576 |
| 4722 | 16.8207 | 12.2335 | 8.18792 | 146.765 | 89.082 | 91.819 |
| 33-16 | 26.2046 | 12.999 | 7.38146 | 217.674 | 87.57 | 89.296 |
| 38-11 | 49.1965 | 17.3515 | 6.36763 | 190.659 | 91.592 | 93.288 |
| A188 | 17.7232 | 8.8113 | 7.46131 | 160.86 | 71.972 | 72.003 |
| A214N | 13.5108 | 11.7649 | 8.00931 | 185.88 | 85.423 | 101.961 |
| A239 | 20.0565 | 12.3342 | 7.38323 | 163.747 | 79.815 | 81.387 |
| A441-5 | 16.3204 | 12.1847 | 7.51154 | 147.342 | 92.316 | 94.233 |
| A554 | 13.2427 | 8.1642 | 7.98556 | 145.265 | 70.854 | 70.85 |
| A556 | 22.0923 | 13.4765 | 8.2564 | 142.622 | 75.486 | 74.945 |
| A6 | 36.7206 | 20.0165 | 7.05069 | 197.155 | 100.296 | 106.958 |
| A619 | 21.1374 | 11.8132 | 6.44854 | 151.415 | 75.181 | 82.425 |
| A632 | 29.8061 | 17.8146 | 7.79421 | 172.502 | 80.625 | 81.109 |
| A634 | 23.9207 | 11.4468 | 7.85808 | 163.709 | 79.228 | 80.287 |
| A635 | 24.9068 | 11.1671 | 7.69506 | 152.226 | 78.046 | 81.41 |
| A641 | 20.2595 | 9.1305 | 7.33361 | 136.445 | 73.146 | 73.769 |
| A654 | 13.3336 | 12.1625 | 7.2912 | 131.348 | 69.431 | 70.54 |
| A659 | 19.3622 | 9.845 | 7.55268 | 182.602 | 78.954 | 80.286 |
| A661 | 13.2877 | 8.4809 | 8.08785 | 160.061 | 69.083 | 72.162 |
| A679 | 23.1871 | 9.8523 | 8.186 | 182.05 | 78.926 | 76.796 |
| A680 | 36.1613 | 15.1434 | 7.85104 | 198.512 | 83.796 | 84.302 |
| A682 | 30.9327 | 14.2141 | 7.4399 | 172.363 | 74.109 | 75.563 |
| AB28A | 17.9183 | 10.9813 | 7.73861 | 175.032 | 93.252 | 95.371 |
| B10 | 18.5698 | 12.8806 | 7.65979 | 134.542 | 91.541 | 96.702 |
| B103 | 22.2203 | 13.9012 | 7.54276 | 144.534 | 78.837 | 80.215 |
| B104 | 9.4813 | 9.0739 | 8.47211 | 149.423 | 86.956 | 88.873 |
| B105 | 27.0718 | 13.1077 | 7.57625 | 178.167 | 83.712 | 85.043 |
| B109 | 30.2277 | 12.553 | 7.73925 | 196.702 | 82.47 | 81.682 |
| B14A | 19.2663 | 8.7273 | 7.88342 | 182.735 | 87.173 | 92.364 |
| B164 | 18.4458 | 6.0571 | 8.36471 | 182.64 | 80.216 | 82.88 |
| B2 | 29.0266 | 11.6194 | 6.57718 | 181.667 | 87.053 | 89.298 |
| B37 | 25.1572 | 11.6085 | 7.51167 | 165.75 | 81.966 | 86.554 |
| B46 | 26.1616 | 12.9571 | 7.32305 | 160.089 | 86.134 | 88.77 |
| B52 | 7.2982 | 5.4741 | 7.56588 | 183.944 | 90.148 | 89.545 |
| B57 | 13.1057 | 8.1444 | 7.31632 | 150.788 | 83.672 | 85.718 |
| B64 | 17.1339 | 9.6842 | 8.4878 | 202.242 | 90.518 | 90.581 |
| B68 | 32.5864 | 20.0014 | 7.38375 | 192.909 | 89.955 | 91.801 |
| B73 | 26.9864 | 14.2443 | 8.31391 | 195.618 | 84.598 | 85.852 |
| B73HTRHM | 32.5142 | 14.555 | 8.55939 | 210.343 | 85.364 | 86.611 |
| B75 | 40.4081 | 17.2553 | 7.68985 | 187.338 | 81.314 | 84.199 |
| B76 | 25.1335 | 12.9071 | 6.81074 | 139.511 | 81.602 | 81.254 |
| B77 | 13.0874 | 10.4725 | 7.90867 | 208.6 | 87.131 | 88.121 |
| B79 | 17.9294 | 8.572 | 7.08262 | 168.74 | 87.963 | 87.972 |
| B84 | 16.5252 | 11.3949 | 7.94036 | 140.567 | 88.442 | 90.318 |
| B97 | 22.1492 | 12.2364 | 7.63742 | 177.972 | 84.467 | 87.508 |
| C103 | 18.2681 | 13.2162 | 7.23378 | 200.448 | 80.741 | 84.834 |
| C123 | 21.2823 | 13.02 | 7.37284 | 160.943 | 74.835 | 76.733 |
| C49A | 11.1522 | 8.9216 | 5.68894 | 129.005 | 66.536 | 69.125 |
| CH701-30 | 25.1395 | 15.0766 | 7.69878 | 175.71 | 75.56 | 80.855 |
| CH9 | 18.3145 | 10.361 | 6.80262 | 160.01 | 80.896 | 85.544 |
| CI.7 | 35.7402 | 14.241 | 7.32581 | 222.072 | 93.565 | 93.342 |
| CI187-2 | 26.3112 | 11.0857 | 7.08093 | 153.389 | 85.303 | 90.519 |
| CI21E | 23.97 | 10.2884 | 7.72645 | 203.65 | 90.13 | 92.263 |
| CI28A | 11.128 | 8.4481 | 7.03817 | 167.407 | 90.596 | 93.446 |
| CI31A | 23.0272 | 8.2893 | 7.93607 | 158.86 | 91.9 | 93.761 |
| CI3A | 28.2764 | 12.354 | 7.52936 | 204.177 | 87.964 | 90.882 |
| CI64 | 28.9111 | 10.2428 | 6.95181 | 193.699 | 95.483 | 93.592 |
| CI66 | 13.1672 | 10.1771 | 7.98252 | 171.646 | 98.434 | 107.296 |
| CI90C | 17.6815 | 12.1607 | 7.65957 | 172.767 | 95.912 | 102.052 |
| CI91B | 15.8966 | 9.5708 | 7.54083 | 143.443 | 87.926 | 91.478 |
| CM105 | 20.6713 | 11.112 | 8.07738 | 144.228 | 71.313 | 72.844 |
| CM174 | 12.5141 | 7.8512 | 7.78155 | 138.4 | 66.672 | 69.315 |
| CM37 | 11.7595 | 14.7777 | 7.20229 | 104.255 | 63.726 | 66.145 |
| CM7 | 12.7997 | 11.2041 | 6.90681 | 114.019 | 61.634 | 65.071 |
| CML10 | 15.1671 | 8.8199 | 8.90596 | 212.455 | 109.4 | 112.163 |
| CML103 | 24.334 | 11.2451 | 8.34671 | 201.275 | 95.137 | 94.503 |
| CML108 | 18.7822 | 9.718 | 7.51628 | 185.711 | 94.342 | 94.771 |
| CML11 | 15.5035 | 8.9849 | 8.76138 | 198.415 | 106.771 | 108.904 |
| CML154Q | 16.2629 | 11.3248 | 7.98896 | 178.672 | 95.786 | 96.839 |
| CML157Q | 15.493 | 9.2086 | 8.93596 | 202.036 | 100.903 | 104.886 |
| CML158Q | 9.7212 | 7.974 | 9.01764 | 189.592 | 102.648 | 106.298 |
| CML218 | 15.1472 | 10.289 | 8.77521 | 210.298 | 99.762 | 102.236 |
| CML220 | 12.8686 | 9.0062 | 7.77483 | 135.857 | 91.585 | 92.285 |
| CML228 | 8.2743 | 8.2691 | 8.5089 | 199.579 | 104.341 | 105.506 |
| CML238 | 14.1446 | 11.4973 | 8.08233 | 168.78 | 96.916 | 98.319 |
| CML247 | 14.3871 | 11.3016 | 8.15969 | 168.641 | 110.21 | 114.355 |
| CML254 | 13.9506 | 16.619 | 9.03805 | - | - | 126.222 |
| CML258 | 22.8338 | 11.299 | 8.99912 | 212.115 | 117.663 | 118.878 |
| CML261 | 19.7251 | 6.0252 | - | 241.079 | 117.967 | 118.475 |
| CML264 | 5.1941 | 6.963 | - | 224.813 | 123.393 | - |
| CML277 | 7.833 | 4.6672 | 9.06222 | 167.876 | 107.805 | 109.128 |
| CML287 | 16.7477 | 9.9376 | 8.73516 | 217.157 | 115.412 | 114.182 |
| CML311 | 18.0887 | 7.4107 | 6.92637 | 187.698 | 100.109 | 104.169 |
| CML314 | 20.7337 | 9.7564 | 7.99969 | 216.502 | 102.191 | 107.247 |
| CML321 | 24.1629 | 12.1386 | 8.40451 | 205.384 | 110.747 | 113.205 |
| CML322 | 18.2611 | 13.8993 | 8.43336 | 185.721 | 94.743 | 96.296 |
| CML323 | 12.8514 | 14.1859 | 8.32811 | 159.151 | 93.269 | 96.929 |
| CML328 | 18.5495 | 11.5112 | 8.02038 | 196.289 | 99.789 | 100.844 |
| CML331 | 18.6355 | 12.3027 | 8.90095 | 235.411 | 112.299 | 118.708 |
| CML332 | 9.6269 | 9.8819 | 7.89798 | 182.323 | 101.311 | 109.072 |
| CML333 | 20.7741 | 11.2931 | 8.72892 | 213.126 | 100.148 | 103.245 |
| CML341 | 7.152 | 8.1112 | 8.94286 | 225.834 | 108.315 | 113.459 |
| CML38 | 19.6047 | 8.7311 | 8.85796 | 195.587 | 106.428 | 107.094 |
| CML45 | 32.9925 | 12.1988 | 8.71128 | 220.028 | 119.06 | 118.94 |
| CML5 | 25.5923 | 14.72 | 8.51531 | 228.142 | 116.309 | 116.034 |
| CML52 | 11.1641 | 14.7911 | 8.44718 | 183.699 | 114.685 | 113.135 |
| CML61 | 14.2124 | 14.593 | 8.91503 | 267.511 | 118.258 | 122.011 |
| CML69 | 19.0828 | 9.3032 | 8.94702 | 201.854 | 101.488 | 104.336 |
| CML77 | 17.0593 | 9.4361 | 7.6921 | 187.71 | 92.297 | 90.717 |
| CML91 | 11.1132 | 5.5533 | 8.6358 | 204.786 | 99.722 | 100.983 |
| CML92 | 17.3502 | 9.3229 | 8.36216 | 211.719 | 94.662 | 94.648 |
| CMV3 | 7.55 | 6.4966 | 6.43964 | 136.797 | 62.837 | 66.369 |
| CO106 | 14.3226 | 8.3862 | 7.64993 | 135.361 | 66.447 | 68.421 |
| CO125 | 19.1522 | 13.0518 | 7.19838 | 133.994 | 70.46 | 71.657 |
| CO255 | 22.9476 | 12.8127 | 7.49144 | 164.937 | 62.955 | 66.009 |
| D940Y | 14.3237 | 8.3086 | 7.61244 | 202.212 | 100.229 | 103.794 |
| DE_2 | 20 | 14.6893 | 7.22953 | 178.858 | 87.355 | 87.339 |
| DE1 | 25.4943 | 8.7876 | 7.31567 | 179.072 | 84.65 | 87.263 |
| DE811 | 16.8254 | 11.3103 | 8.02255 | 184.348 | 89.474 | 88.311 |
| E2558W | 34.9465 | 15.3759 | 7.81572 | 192.88 | 89.847 | 93.886 |
| EP1 | 23.05 | 10.6757 | 6.53056 | 135.371 | 65.678 | 70.107 |
| F2834T | 20.1096 | 9.4024 | 8.13925 | 179.767 | 86.984 | 88.627 |
| F44 | 23.1798 | 14.5819 | 8.21071 | 219.92 | 99.763 | 100.601 |
| F6 | 20.556 | 15.7327 | 8.08298 | 201.526 | 100.456 | 101.872 |
| F7 | 13.9815 | 11.1145 | 7.23163 | 116.13 | 62.778 | 66.102 |
| GA209 | 11.7738 | 9.9856 | 8.07499 | 189.291 | 93.945 | 95.714 |
| GT112 | 27.142 | 15.2451 | 7.37593 | 206.409 | 100.977 | 103.848 |
| H105W | 27.2979 | 16.5718 | 7.97375 | 179.655 | 81.002 | 83.319 |
| H49 | 42.1541 | 22.6233 | 6.41664 | 174.076 | 89.013 | 89.679 |
| H84 | 20.6768 | 12.6465 | 7.68322 | 169.944 | 80.738 | 86.093 |
| H91 | 25.5844 | 14.3321 | 7.89654 | 186.385 | 84.184 | 85.799 |
| H95 | 18.2054 | 10.2275 | 7.78403 | 172.713 | 85.945 | 87.889 |
| H99 | 16.6278 | 11.4543 | 7.37805 | 116.945 | 77.28 | 77.333 |
| HI27 | 18.9983 | 10.166 | 8.10353 | 198.417 | 97.263 | 100.567 |
| HP301 | 34.6086 | 17.9938 | 8.21298 | 178.037 | 81.132 | 83.033 |
| HY | 34.5769 | 13.6401 | 7.34864 | 199.119 | 83.216 | 87.244 |
| I137TN | 19.7975 | 14.2483 | 8.54049 | 167.905 | 92.679 | 95.259 |
| I205 | 12.4667 | 5.1533 | 7.61628 | 147.511 | 85.01 | 87.772 |
| I29 | 13.3753 | 10.6607 | 7.64744 | 148.599 | 70.818 | 70.622 |
| IA2132 | 19.6326 | 10.9005 | 7.76039 | 141.195 | 74.458 | 75.212 |
| IDS28 | 16.2092 | 6.7794 | 7.3706 | 183.501 | 83.976 | 86.506 |
| IDS69 | 21.238 | 13.2301 | 7.27952 | 125.587 | 83.055 | 85.315 |
| IDS91 | 19.5497 | 14.6679 | 7.33989 | 168.134 | 80.5 | 84.884 |
| IL101 | 13.568 | 13.0937 | 5.39723 | 124.953 | 71.663 | 74.484 |
| IL14H | 18.4073 | 7.9277 | 7.79846 | 190.622 | 78.792 | 80.575 |
| IL677A | 20.2947 | 12.4522 | 6.29605 | 157.194 | 85.388 | 90.659 |
| K148 | 10.7103 | 6.8283 | 8.02735 | 185.23 | 88.074 | 92.068 |
| K4 | 19.2957 | 10.9482 | 7.84926 | 181.727 | 93.171 | 96.688 |
| K55 | 28.1531 | 17.4845 | 7.21411 | 160.806 | 85.945 | 87.093 |
| K64 | 27.4349 | 14.3206 | 7.58619 | 203.604 | 93.758 | 96.107 |
| KI11 | 11.1875 | 11.4084 | 8.61272 | 155.568 | 104.352 | 109.408 |
| KI14 | 35.7084 | 14.7611 | 8.26444 | 202.309 | 110.01 | 119.134 |
| KI2021 | 38.3143 | 19.6235 | 8.36528 | 194.018 | 99.34 | 101.264 |
| KI21 | 28.7852 | 16.9619 | 8.317 | 197.769 | 95.364 | 95.325 |
| KI3 | 8.7235 | 8.0453 | 8.89648 | 167.706 | 98.099 | 101.755 |
| KI43 | 7.8758 | 10.9737 | 8.29393 | 158.593 | 96.813 | 96.47 |
| KI44 | 6.6773 | 7.304 | 8.84519 | 164.939 | 104.04 | 109.126 |
| KY21 | 28.9402 | 12.6343 | 8.03659 | 176.692 | 90.516 | 93.062 |
| KY226 | 29.8443 | 15.9142 | 5.88254 | 208.348 | 93.232 | 94.087 |
| KY228 | 34.4151 | 10.1914 | 7.19858 | 181.564 | 92.785 | 96.678 |
| L317 | 28.5372 | 9.8867 | 6.81542 | 197.308 | 86.673 | 93.956 |
| L578 | 23.3649 | 9.8704 | 8.33726 | 223.061 | 95.617 | 96.505 |
| M14 | 24.9753 | 9.7332 | 7.75477 | 150.422 | 81.172 | 81.948 |
| M162W | 25.0653 | 12.7057 | 8.01703 | 174.219 | 95.78 | 96.288 |
| M37W | 25.0035 | 14.1284 | 7.89564 | 188.29 | 89.749 | 92.132 |
| MO17 | 29.7182 | 14.7759 | 7.88707 | 197.464 | 79.993 | 83.408 |
| MO18W | 31.9731 | 18.4847 | 7.8105 | 195.544 | 100.687 | 112.435 |
| MO1W | 20.3186 | 13.3185 | 7.34476 | 177.667 | 85.361 | 89.465 |
| MO24W | 31.2211 | 10.5432 | 7.44438 | 226.869 | 88.513 | 92.633 |
| MO44 | 39.5754 | 16.8564 | 7.70625 | 205.212 | 85.279 | 88.611 |
| MO45 | 22.412 | 14.6867 | 8.14905 | 191.61 | 84.155 | 83.968 |
| MO46 | 15.1463 | 9.8583 | 8.11853 | 180.315 | 80.916 | 85.135 |
| MO47 | 21.6358 | 11.0691 | 7.1731 | 156.019 | 79.521 | 81 |
| MOG | 37.2273 | 14.3507 | 8.06248 | 221.972 | 92.221 | 92.564 |
| MP339 | 32.4083 | 14.5761 | 8.37056 | 184.234 | 96.489 | 100.991 |
| MS1334 | 20.5498 | 9.9902 | 7.043 | 169.694 | 70.313 | 71.252 |
| MS153 | 27.176 | 12.4741 | 7.76106 | 181.696 | 70.911 | 72.389 |
| MS71 | 20.6652 | 13.0321 | 7.28939 | 167.97 | 78.505 | 80.582 |
| MT42 | 10.9524 | 10.6125 | 7.68318 | 129.559 | 65.852 | 69.379 |
| N192 | 24.6245 | 12.9768 | 8.09703 | 159.849 | 76.919 | 78.199 |
| N28HT | 18.4849 | 11.8455 | 7.6667 | 100.995 | 87.658 | 88.756 |
| N6 | 22.0506 | 11.3572 | 7.74028 | 155.818 | 81.006 | 84.504 |
| N7A | 21.0311 | 9.7779 | 8.21063 | 164.076 | 82.154 | 82.301 |
| NC222 | 18.5131 | 8.2642 | 8.57036 | 189.852 | 96.191 | 100.933 |
| NC230 | 13.0283 | 7.1255 | 8.34776 | 153.347 | 91.014 | 93.026 |
| NC232 | 27.9829 | 18.5236 | 8.03155 | 170.027 | 90.078 | 91.933 |
| NC236 | 23.5254 | 14.7685 | 6.9796 | 156.788 | 95.027 | 94.32 |
| NC250 | 24.3761 | 18.1527 | 7.78544 | 128.94 | 84.041 | 85.763 |
| NC258 | 15.4299 | 7.3275 | 8.71987 | 187.917 | 90.916 | 91.951 |
| NC260 | 15.8778 | 5.7828 | 7.73022 | 200.372 | 84.231 | 86.448 |
| NC262 | 16.733 | 9.8848 | 8.1774 | 161.424 | 76.12 | 79.095 |
| NC264 | 14.1189 | 11.0928 | 8.59522 | 171.555 | 86.412 | 88.123 |
| NC290A | 20.1931 | 11.2508 | 7.85487 | 175.142 | 84.828 | 87.428 |
| NC294 | 21.8972 | 15.2657 | 8.67092 | 178.141 | 85.787 | 87.971 |
| NC296 | 18.3118 | 9.2259 | 8.37963 | 196.215 | 95.353 | 95.338 |
| NC296A | 11.7641 | 9.6102 | 8.71807 | 175.38 | 94.435 | 97.105 |
| NC298 | 15.4946 | 14.1304 | 8.04565 | 166.292 | 92.74 | 94.884 |
| NC300 | 13.4399 | 7.1667 | 7.95708 | 181.792 | 102.58 | 104.193 |
| NC302 | 24.4111 | 13.5911 | 8.68587 | 167.101 | 89.907 | 92.858 |
| NC304 | 12.8378 | 13.3345 | 8.38593 | 118.932 | 93.656 | 96.383 |
| NC306 | 35.2023 | 19.9578 | 7.94656 | 185.44 | 88.247 | 88.106 |
| NC310 | 19.08 | 9.712 | 8.31023 | 173.981 | 83.18 | 85.871 |
| NC314 | 18.8969 | 13.1373 | 6.60756 | 131.318 | 83.092 | 87.019 |
| NC318 | 21.9555 | 10.8465 | 8.96895 | 199.98 | 94.78 | 92.929 |
| NC320 | 19.8541 | 9.9611 | 8.32209 | 208.752 | 94.298 | 95.548 |
| NC324 | 25.6865 | 21.4253 | 7.2274 | 133.697 | 83.698 | 88.413 |
| NC326 | 29.2424 | 13.3168 | 8.04252 | 184.086 | 84.538 | 87.352 |
| NC328 | 30.9296 | 14.1602 | 8.53527 | 192.114 | 88.45 | 88.363 |
| NC33 | 33.2204 | 12.6508 | 7.70486 | 245.338 | 97.496 | 103.988 |
| NC336 | 15.4097 | 8.4205 | 8.16503 | 177.722 | 99.631 | 101.877 |
| NC338 | 13.2464 | 10.1582 | 8.28913 | 173.457 | 95.147 | 96.647 |
| NC340 | 18.9835 | 11.376 | 8.4835 | 190.497 | 96.112 | 96.545 |
| NC342 | 30.9853 | 16.36 | 8.18227 | 193.253 | 87.41 | 88.088 |
| NC344 | 11.0991 | 8.4625 | 8.21732 | 206.885 | 90.249 | 90.018 |
| NC346 | 6.6627 | 6.9189 | 8.39271 | 187.339 | 92.82 | 94.922 |
| NC348 | 8.9365 | 4.1024 | 8.56422 | 189.852 | 90.49 | 90.924 |
| NC350 | 20.2647 | 11.185 | 8.64909 | 157.401 | 93.903 | 96.235 |
| NC352 | 12.7732 | 9.5831 | 8.57111 | 188.515 | 93.696 | 97.079 |
| NC354 | 23.1902 | 10.6721 | 7.67331 | 179.914 | 92.517 | 93.112 |
| NC356 | 11.9551 | 14.401 | 8.44665 | 166.925 | 91.713 | 93.127 |
| NC358 | 12.0061 | 8.9743 | 8.52556 | 164.71 | 86.342 | 87.593 |
| NC360 | 22.9774 | 15.0578 | 7.95591 | 163.523 | 90.519 | 89.162 |
| NC362 | 34.7526 | 16.3899 | 7.21687 | 194.583 | 86.115 | 86.844 |
| NC364 | 28.2796 | 19.9071 | 8.01819 | 156.62 | 80.298 | 79.443 |
| NC366 | 12.8534 | 8.2127 | 8.06499 | 183.541 | 98.465 | 98.761 |
| NC368 | 21.9325 | 10.353 | 7.66021 | 192.808 | 88.016 | 89.165 |
| ND246 | 14.1171 | 9.5538 | 7.91858 | 124.177 | 63.962 | 70.952 |
| OH40B | 13.8343 | 13.8254 | 7.29239 | 169.856 | 80.151 | 84.738 |
| OH43 | 22.6883 | 14.712 | 7.43736 | 166.139 | 78.372 | 81.844 |
| OH43E | 11.1951 | 9.3632 | 8.04956 | 143.594 | 77.886 | 80.513 |
| OH603 | 40.4131 | 19.0483 | 7.03112 | 189.638 | 85.05 | 85.702 |
| OH7B | 19.9555 | 6.1717 | 8.24706 | 224.075 | 91.706 | 95.391 |
| P39 | 13.3192 | 12.1152 | 6.25411 | 128.203 | 70.404 | 72.817 |
| PA762 | 34.2379 | 14.85 | 8.28094 | 168.83 | 83.985 | 85.379 |
| PA875 | 23.7211 | 19.8058 | 7.16358 | 152.215 | 83.853 | 85.85 |
| PA880 | 14.204 | 9.1047 | 7.51423 | 194.843 | 85.222 | 86.388 |
| PA91 | 21.6097 | 10.8437 | 7.28854 | 213.176 | 85.39 | 87.587 |
| R109B | 14.7991 | 7.539 | 7.58125 | 174.715 | 80.156 | 82.375 |
| R168 | 14.0408 | 6.6613 | 7.94507 | 170.197 | 81.566 | 80.278 |
| R229 | 31.3388 | 14.5602 | 7.86256 | 188.245 | 86.986 | 88.381 |
| R4 | 20.1758 | 10.5362 | 6.99043 | 189.626 | 86.531 | 91.025 |
| SA24 | 18.9038 | 14.9734 | 8.05869 | 171.505 | 87.019 | 87.301 |
| SC213R | 15.7293 | 14.344 | 8.43457 | 189.158 | 105.274 | 108.862 |
| SC357 | 20.8394 | 10.3639 | 8.14132 | 184.943 | 95.193 | 94.027 |
| SC55 | 22.5697 | 11.0488 | 8.21433 | 205.76 | 103.553 | 106.575 |
| SD40 | 25.1812 | 15.5561 | 8.26122 | 154.444 | 81.11 | 84.395 |
| SD44 | 20.4163 | 12.3283 | 7.56867 | 191.768 | 74.95 | 76.772 |
| SG1533 | 33.6532 | 16.5704 | 8.70873 | 188.631 | 86.342 | 86.861 |
| SG18 | 26.0916 | 11.3164 | 7.4221 | 201.315 | 82.456 | 84.629 |
| T232 | 25.0192 | 13.3988 | 8.07575 | 212.439 | 90.408 | 92.103 |
| T234 | 24.637 | 10.377 | 8.10833 | 178.244 | 90.811 | 94.667 |
| T8 | 21.7562 | 11.0004 | 7.95939 | 202.179 | 91.469 | 91.218 |
| TX303 | 37.7041 | 15.0909 | 7.90143 | 223.813 | 97.458 | 101.187 |
| TX601 | 31.6689 | 18.3657 | 8.65351 | 210.232 | 109.123 | 109.74 |
| TZI10 | 20.4861 | 11.5901 | 8.94585 | 222.554 | 110.934 | 117.694 |
| TZI11 | 14.4385 | 8.7107 | 7.93428 | 202.231 | 96.857 | 99.295 |
| TZI16 | 17.7499 | 6.6478 | 8.49951 | 231.488 | 95.949 | 92.959 |
| TZI18 | 20.8219 | 11.7956 | 8.45052 | 200.754 | 100.185 | 101.218 |
| TZI25 | 17.5515 | 12.2052 | 8.02647 | 200.434 | 91.594 | 93.677 |
| TZI9 | 16.4365 | 10.2275 | 7.56252 | 213.312 | 94.084 | 93.387 |
| U267Y | 23.6972 | 14.183 | 6.84665 | 177.828 | 94.629 | 99.017 |
| VA102 | 24.0311 | 12.9724 | 6.89367 | 178.437 | 80.461 | 86.841 |
| VA14 | 19.8686 | 11.6397 | 7.73507 | 192.946 | 91.385 | 94.66 |
| VA17 | 22.8301 | 9.6631 | 7.9562 | 161.546 | 88.523 | 92.988 |
| VA22 | 24.2627 | 15.0942 | 7.77701 | 182.607 | 86.98 | 88.099 |
| VA26 | 13.4072 | 12.2725 | 8.03426 | 199.566 | 82.026 | 83.22 |
| VA35 | 16.5473 | 8.46 | 7.76434 | 177.919 | 84.031 | 84.992 |
| VA59 | 22.6627 | 12.4285 | 8.09831 | 173.805 | 78.09 | 81.171 |
| VA85 | 24.956 | 18.709 | 7.08505 | 207.131 | 85.388 | 87.247 |
| VA99 | 30.601 | 14.8028 | 7.63232 | 198.97 | 82.726 | 86.438 |
| VAW6 | 17.644 | 12.59 | 7.52731 | 136.507 | 83.034 | 86.221 |
| W117HT | 12.177 | 8.895 | 7.42496 | 109.933 | 70.089 | 72.823 |
| W153R | 12.6741 | 9.3028 | 6.62854 | 115.477 | 75.097 | 76.773 |
| W182B | 23.4151 | 16.7702 | 7.87471 | 148.463 | 66.814 | 68.76 |
| W22 | 20.0697 | 16.1913 | 7.98525 | 158.886 | 84.107 | 86.104 |
| WF9 | 35.7291 | 19.4377 | 6.58862 | 181.717 | 83.728 | 84.7 |
| YU796 | 12.6968 | 9.1375 | 7.86385 | 149.116 | 79.418 | 80.624 |

TL, tunnel length; SD, stem damage; KR, kernel resistance; PH, plant height; DTA, days to anthesis; DTS, days to silking.

**Table S4 Annual and average means and range of variation for traits related to resistance to MCB and three agronomic traits.**

|  |  | Resistance traits | | |  | Agronomic traits | | |
| --- | --- | --- | --- | --- | --- | --- | --- | --- |
| Environment | Estimate | TL  (cm) | SD  (%) | KR  (1-9)^†^ |  | PH  (cm) | DTA  (days) | DTS  (days) |
| 2010 | Mean | 17.5^‡^ | 9.9 | 7.6 |  | 180.6 | 79.6 | 81.5 |
|  | Range | 1.6-54.8 | 1.1-30.3 | 4.8-9.1 |  | 106.6-250.7 | 56.6-115.1 | 57-116.5 |
| 2011 | Mean | 14.7 | 8.7 | 8.1 |  | 169.7 | 92.6 | 94.7 |
|  | Range | 0.3-39.9 | 2.0-21.9 | 3.4-9.0 |  | 91.7-257.5 | 61.6-127.7 | 66.6-126-7 |
| 2012 | Mean | 43.3 | 22.7 | 7.7 |  | 182.1 | 92.1 | 93.9 |
|  | Range | 6.1-96.9 | 3.3-46.7 | 4.6-9.3 |  | 92.2-253 | 56.2-122.0 | 68.3-122.6 |
| Combined | Mean | 20.9 | 11.9 | 7.8 |  | 178.2 | 88.5 | 90.7 |
|  | Range | 5.2-49.2 | 4.1-22.6 | 5.4-9 |  | 101-267.5 | 61.6-123.4 | 65.1-126.2 |

TL, tunnel length; SD, stem damage; KR, kernel resistance, PH, plant height; DTA, day to anthesis; DTS, day to silking.

^†^ Ear appearance was estimated on a subjective visual scale of 1 to 9 in which 1 indicates completely damaged and 9 indicates no damage.

^‡^Mean values with different letter were significantly different according to LSD (α = 0.05)

**Table S5 – Summary of the SNPs significantly associated to plant height.** SNP identification (SNP ID), additive effect and allelic variants for the SNP, proportion of total variance explained by the SNPs significantly associated with plant height (PH) and significance values for the association between the SNP and the phenotype (*P*-value and RMIP).

| Trait | SNP ID^a^ | Alleles^b^ | | (N^o^)^c^ | AdditiveEffect^d^ | *P*-value | (*R*^2^)^e^ | RMIP^f^ |
| --- | --- | --- | --- | --- | --- | --- | --- | --- |
| PH | S1_77272761 | C/G | 242/22 | | -13.00 | 6.51 × 10^-5^ | 0.06 | 0.30 |
| PH | S1_243506545 | C/G | 221/26 | | 13.05 | 1.22 × 10^-5^ | 0.08 | 0.41 |
| PH | S1_271903514 | T/C | 150/104 | | 8.20 | 9.21 × 10^-6^ | 0.08 | 0.40 |
| PH | S2_3990193 | C/A | 158/103 | | 7.90 | 4.84 × 10^-6^ | 0.08 | 0.51 |
| PH | S2_28000786 | G/T | 253/12 | | 16.20 | 1.11 × 10^-5^ | 0.08 | 0.58 |
| PH | S2_105720343 | C/T | 239/24 | | -11.55 | 5.56 × 10^-5^ | 0.06 | 0.56 |
| PH | S3_127931464 | A/C | 186/67 | | 8.60 | 1.36 × 10^-5^ | 0.08 | 0.40 |
| PH | S3_127931465 | G/A | 186/67 | | 8.60 | 1.36 × 10^-5^ | 0.08 | 0.40 |
| PH | ss3_156639988^g^ | C/A | 219/45 | | -8.85 | 6.10 × 10^-5^ | 0.06 | 0.38 |
| PH | S3_156788803 | C/T | 224/37 | | -9.40 | 1.13 × 10^-4^ | 0.06 | 0.46 |
| PH | ss3_157051848^g^ | A/C | 128/128 | | -7.75 | 1.90 × 10^-6^ | 0.09 | 0.70 |
| PH | S4_220906905 | G/A | 182/72 | | -8.70 | 4.71 × 10^-5^ | 0.07 | 0.35 |
| PH | S5_1527023 | T/C | 238/21 | | -11.80 | 6.76 × 10^-5^ | 0.06 | 0.31 |
| PH | S6_5301001 | G/A | 217/31 | | 10.8 | 2.74 × 10^-5^ | 0.07 | 0.36 |
| PH | S6_79550091 | C/G | 158/89 | | -8.45 | 1.26 × 10^-5^ | 0.08 | 0.45 |
| PH | S6_80646742 | T/C | 169/93 | | 7.15 | 3.16 × 10^-5^ | 0.07 | 0.45 |
| PH | S9_20470625 | C/T | 217/13 | | 15.75 | 1.47 × 10^-5^ | 0.09 | 0.57 |
| PH | S9_142752809 | A/G | 201/43 | | -8.70 | 4.00 × 10^-5^ | 0.07 | 0.34 |

^a^ The number before the underscore (_) indicates the chromosome number and the number after the underscore (_) indicates the physical position in bp within the chromosome .

^b^ The letter before the diagonal (/) is the nucleotide more frequent ; and the letter after the diagonal the nucleotide less frequent.

^c^ Nº = number of inbred lines homozygous for a determined allelic variant. The number before the diagonal (/) represents the number of individuals with the mayor allele; and the number after the diagonal represents the number of individuals with the minor allele.

^d^ The additive effect was calculated as half the difference between the mean of the homozygous for the minor and the mean of the homozygous for the major allele. It is given in centimeters.

^e^ *R^2^*, proportion of the phenotypic variance explained by the SNP .

^f^ RMIP, resample model inclusion probability.

^g^ Based on SNPs from Illumina chip, the remaining locations without a superscript are based on SNPs obtained by GBS.

**Table S6 – Summary of the SNPs significantly associated to days to anthesis.** SNP identification (SNP ID), additive effect and allelic variants for the SNP, proportion of total variance explained by the SNPs significantly associated with days to anthesis (DTA) and significance values for the association between the SNP and the phenotype (*P*-value and RMIP).

| Trait | SNP ID^a^ | Alleles^b^ | | (N^o^)^c^ | AdditiveEffect^d^ | *P*-value | (*R*^2^)^e^ | RMIP^f^ |
| --- | --- | --- | --- | --- | --- | --- | --- | --- |
| DTA | S1_208762624 | C/T | 231/29 | | 3.70 | 1.13 × 10^-5^ | 0.08 | 0.52 |
| DTA | S1_237922464 | C/T | 191/52 | | 2.70 | 7.20 × 10^-5^ | 0.07 | 0.36 |
| DTA | S1_268434937 | G/C | 204/30 | | 3.80 | 1.19 × 10^-5^ | 0.08 | 0.78 |
| DTA | ss1_271970014^g^ | A/C | 244/21 | | -3.90 | 6.37 × 10^-5^ | 0.06 | 0.30 |
| DTA | S1_272275112 | G/A | 244/19 | | -4.10 | 5.14 × 10^-5^ | 0.06 | 0.35 |
| DTA | S1_295982332 | C/T | 223/26 | | 3.65 | 7.26 × 10^-6^ | 0.08 | 0.71 |
| DTA | S2_13616629 | C/G | 239/6 | | -6.75 | 3.20 × 10^-5^ | 0.07 | 0.50 |
| DTA | S3_158897644 | T/C | 162/82 | | -3.10 | 1.62 × 10^-8^ | 0.14 | 0.99 |
| DTA | S3_159389461 | C/G | 166/80 | | -2.75 | 9.22 × 10^-7^ | 0.11 | 0.73 |
| DTA | S3_159389463 | C/A | 174/70 | | -3.05 | 1.25 × 10^-7^ | 0.12 | 0.99 |
| DTA | S3_159555813 | C/A | 185/66 | | -2.90 | 3.97 × 10^-7^ | 0.11 | 0.94 |
| DTA | S3_161702976 | G/T | 197/42 | | 3.05 | 4.60 × 10^-6^ | 0.09 | 0.53 |
| DTA | S3_161702979 | T/A | 197/42 | | 3.05 | 4.60 × 10^-6^ | 0.09 | 0.53 |
| DTA | S4_5466142 | T/C | 128/116 | | 2.05 | 8.94 × 10^-5^ | 0.06 | 0.35 |
| DTA | S4_15583016 | A/T | 119/115 | | -2.45 | 1.63 × 10^-5^ | 0.08 | 0.53 |
| DTA | ss4_44749825^g^ | G/A | 224/39 | | 3.45 | 5.20 × 10^-5^ | 0.06 | 0.51 |
| DTA | ss4_44751539^g^ | C/A | 226/39 | | 3.45 | 5.30 × 10^-5^ | 0.06 | 0.50 |
| DTA | S4_213549036 | A/G | 246/12 | | 6.15 | 1.38 × 10^-5^ | 0.08 | 0.64 |
| DTA | S5_175211216 | T/C | 172/69 | | -2.20 | 7.22 × 10^-5^ | 0.07 | 0.32 |
| DTA | S5_175211217 | G/A | 172/69 | | -2.20 | 7.22 × 10^-5^ | 0.07 | 0.32 |
| DTA | S5_175211218 | T/C | 172/69 | | -2.20 | 7.22 × 10^-5^ | 0.07 | 0.32 |
| DTA | S5_175211219 | T/A | 172/69 | | -2.20 | 7.22 × 10^-5^ | 0.07 | 0.32 |
| DTA | S5_176047685 | A/C | 209/44 | | 2.95 | 3.80 × 10^-5^ | 0.07 | 0.40 |
| DTA | S5_176047686 | G/T | 209/44 | | 2.95 | 3.80 × 10^-5^ | 0.07 | 0.40 |
| DTA | S5_176047687 | C/A | 209/44 | | 2.95 | 3.80 × 10^-5^ | 0.07 | 0.40 |
| DTA | S5_176047690 | A/G | 207/44 | | 3.00 | 3.29 × 10^-5^ | 0.07 | 0.47 |
| DTA | S5_176047703 | A/G | 209/44 | | 2.95 | 3.80 × 10^-5^ | 0.07 | 0.40 |
| DTA | S5_176047734 | G/C | 209/44 | | 2.95 | 3.80 × 10^-5^ | 0.07 | 0.40 |
| DTA | ss6_81693857^g^ | A/C | 243/16 | | 4.45 | 5.47 × 10^-5^ | 0.06 | 0.31 |
| DTA | S8_123506087 | C/G | 168/86 | | -2.35 | 6.50 × 10^-6^ | 0.08 | 0.65 |
| DTA | S8_123506089 | A/T | 168/86 | | -2.35 | 6.50 × 10^-6^ | 0.08 | 0.65 |
| DTA | S8_123506141 | C/T | 168/86 | | -2.35 | 6.50 × 10^-6^ | 0.08 | 0.65 |
| DTA | ss8_123507284^g^ | A/C | 169/97 | | -2.25 | 7.30 × 10^-6^ | 0.08 | 0.61 |
| DTA | S8_123509373 | G/C | 165/94 | | -2.30 | 6.16 × 10^-6^ | 0.08 | 0.61 |
| DTA | S8_130796873 | T/G | 223/39 | | -3.05 | 7.24 × 10^-6^ | 0.08 | 0.59 |
| DTA | S8_131176630 | C/T | 210/39 | | -3.10 | 7.10 × 10^-6^ | 0.09 | 0.51 |
| DTA | S8_131176643 | G/A | 210/46 | | -2.55 | 2.98 × 10^-5^ | 0.07 | 0.55 |
| DTA | S8_132047205 | G/A | 218/30 | | -3.50 | 4.45 × 10^-5^ | 0.07 | 0.46 |
| DTA | S8_132199932 | G/A | 213/44 | | -2.50 | 9.55 × 10^-5^ | 0.06 | 0.40 |
| DTA | S8_132201362 | T/C | 201/57 | | -2.45 | 4.31 × 10^-5^ | 0.07 | 0.60 |
| DTA | S8_132201389 | C/T | 212/46 | | -2.50 | 8.88 × 10^-5^ | 0.06 | 0.37 |
| DTA | S8_132328071 | C/T | 205/47 | | -2.55 | 5.64 × 10^-5^ | 0.07 | 0.48 |
| DTA | S9_128723807 | G/T | 208/43 | | 3.10 | 3.41 × 10^-5^ | 0.07 | 0.60 |
| DTA | S9_144881095 | C/A | 209/32 | | 3.00 | 3.11 × 10^-5^ | 0.08 | 0.31 |
| DTA | S9_144881314 | C/T | 222/31 | | 3.15 | 1.22 × 10^-5^ | 0.08 | 0.46 |
| DTA | S9_147640561 | T/G | 236/17 | | 3.95 | 7.02 × 10^-5^ | 0.07 | 0.33 |
| DTA | S10_34232746 | T/C | 177/80 | | 2.60 | 2.93 × 10^-5^ | 0.07 | 0.52 |
| DTA | S10_148435462 | T/C | 202/58 | | 2.40 | 1.29 × 10^-4^ | 0.06 | 0.31 |

^a^ The number before the underscore (_) indicates the chromosome number and the number after the underscore (_) indicates the physical position in bp within the chromosome .

^b^ The letter before the diagonal (/) is the nucleotide more frequent ; and the letter after the diagonal the nucleotide less frequent.

^c^ Nº = number of inbred lines homozygous for a determined allelic variant. The number before the diagonal (/) represents the number of individuals with the mayor allele; and the number after the diagonal represents the number of individuals with the minor allele.

^d^ The additive effect was calculated as half the difference between the mean of the homozygous for the minor and the mean of the homozygous for the major allele. It is given in days.

^e^ *R^2^*, proportion of the phenotypic variance explained by the SNP .

^f^ RMIP, resample model inclusion probability.

^g^ Based on SNPs from Illumina chip, the remaining locations without a superscript are based on SNPs obtained by GBS.

**Table S7 – Summary of the SNPs significantly associated to days to silking.** SNP identification (SNP ID), additive effect and allelic variants for the SNP, proportion of total variance explained by the SNPs significantly associated with days to silking (DTS) and significance values for the association between the SNP and the phenotype (*P*-value and RMIP).

| Trait | SNP ID^a^ | Alleles^b^ | | (N^o^)^c^ | Additive  Effect^d^ | *P*-value | (*R*^2^)^e^ | RMIP^f^ |
| --- | --- | --- | --- | --- | --- | --- | --- | --- |
| DTS | ss1_271970014^g^ | A/C | 244/21 | | -4.15 | 5.95 × 10^-5^ | 0.06 | 0.39 |
| DTS | S1_272275112 | G/A | 244/19 | | -4.30 | 6.29 × 10^-5^ | 0.06 | 0.33 |
| DTS | S1_295982332 | C/T | 224/25 | | 3.80 | 1.38 × 10^-5^ | 0.08 | 0.58 |
| DTS | S2_41889238 | A/C | 242/15 | | 4.25 | 5.63 × 10^-5^ | 0.07 | 0.35 |
| DTS | S3_158897644 | T/C | 162/82 | | -3.00 | 2.03 × 10^-7^ | 0.12 | 0.96 |
| DTS | S3_159389461 | C/G | 166/80 | | -2.70 | 8.85 × 10^-6^ | 0.09 | 0.56 |
| DTS | S3_159389463 | C/A | 174/70 | | -2.90 | 2.11 × 10^-6^ | 0.10 | 0.80 |
| DTS | S3_159555813 | C/A | 185/66 | | -2.85 | 3.26 × 10^-6^ | 0.09 | 0.84 |
| DTS | S3_159610878 | G/C | 125/117 | | -2.35 | 7.90 × 10^-6^ | 0.08 | 0.63 |
| DTS | S3_159610899 | A/G | 133/117 | | -2.15 | 1.70 × 10^-5^ | 0.08 | 0.52 |
| DTS | S3_159610900 | A/C | 133/117 | | -2.15 | 1.70 × 10^-5^ | 0.08 | 0.52 |
| DTS | S3_159610920 | G/A | 133/117 | | -2.15 | 1.70 × 10^-5^ | 0.08 | 0.52 |
| DTS | S3_161702976 | G/T | 197/42 | | 3.35 | 3.56 × 10^-6^ | 0.09 | 0.73 |
| DTS | S3_161702979 | T/A | 197/42 | | 3.35 | 3.56 × 10^-6^ | 0.09 | 0.73 |
| DTS | S4_5466142 | T/C | 128/116 | | 1.90 | 5.81 × 10^-4^ | 0.05 | 0.31 |
| DTS | S4_5466378 | G/C | 139/101 | | 2.30 | 6.14 × 10^-5^ | 0.07 | 0.33 |
| DTS | S4_237030511 | C/A | 213/31 | | 3.70 | 5.32 × 10^-5^ | 0.07 | 0.35 |
| DTS | S5_174408639 | A/C | 232/17 | | 4.30 | 4.11 × 10^-5^ | 0.07 | 0.30 |
| DTS | S5_175211216 | T/C | 172/69 | | -2.45 | 3.70 × 10^-5^ | 0.07 | 0.45 |
| DTS | S5_175211217 | G/A | 172/69 | | -2.45 | 3.70 × 10^-5^ | 0.07 | 0.45 |
| DTS | S5_175211218 | T/C | 172/69 | | -2.45 | 3.70 × 10^-5^ | 0.07 | 0.45 |
| DTS | S5_175211219 | T/A | 172/69 | | -2.45 | 3.70 × 10^-5^ | 0.07 | 0.45 |
| DTS | S6_7124668 | C/G | 228/20 | | 3.80 | 8.95 × 10^-6^ | 0.08 | 0.49 |
| DTS | S6_36001139 | G/A | 229/27 | | 3.70 | 3.40 × 10^-5^ | 0.07 | 0.37 |
| DTS | S6_70572080 | A/G | 164/86 | | 2.20 | 7.88 × 10^-5^ | 0.06 | 0.33 |
| DTS | ss6_104859110^g^ | A/G | 194/70 | | 3.50 | 1.51 × 10^-5^ | 0.07 | 0.34 |
| DTS | ss6_104865093^g^ | A/G | 195/68 | | 2.95 | 1.75 × 10^-5^ | 0.07 | 0.39 |
| DTS | S6_139199401 | C/A | 246/19 | | 5.20 | 6.27 × 10^-6^ | 0.08 | 0.77 |
| DTS | S6_139199437 | G/A | 246/19 | | 5.20 | 6.27 × 10^-6^ | 0.08 | 0.77 |
| DTS | S6_139212559 | T/G | 246/19 | | 4.70 | 2.99 × 10^-5^ | 0.07 | 0.51 |
| DTS | S7_152699257 | T/G | 185/54 | | -2.35 | 4.45 × 10^-5^ | 0.07 | 0.41 |
| DTS | S8_130796873 | T/G | 223/39 | | -3.30 | 5.12 × 10^-6^ | 0.08 | 0.72 |
| DTS | S8_131176630 | C/T | 210/39 | | -3.45 | 3.01 × 10^-6^ | 0.09 | 0.71 |
| DTS | S8_131176643 | G/A | 210/46 | | -2.60 | 5.91 × 10^-5^ | 0.07 | 0.48 |
| DTS | ss8_131981829^g^ | G/A | 195/66 | | -2.40 | 9.32 × 10^-5^ | 0.06 | 0.31 |
| DTS | S8_132047205 | G/A | 218/30 | | -4.00 | 1.60 × 10^-5^ | 0.08 | 0.6 |
| DTS | S8_162964995 | A/C | 226/37 | | 2.75 | 3.49 × 10^-5^ | 0.07 | 0.33 |
| DTS | S9_128723807 | G/T | 208/43 | | 3.30 | 3.78 × 10^-5^ | 0.07 | 0.57 |
| DTS | S10_34232746 | T/C | 177/80 | | 2.90 | 1.76 × 10^-5^ | 0.07 | 0.53 |
| DTS | S10_34232747 | T/C | 180/78 | | 2.90 | 2.12 × 10^-5^ | 0.07 | 0.48 |
| DTS | S10_34232749 | C/A | 180/78 | | 2.80 | 4.27 × 10^-5^ | 0.07 | 0.38 |
| DTS | ss10_148435462^g^ | A/G | 202/62 | | 2.70 | 5.83 × 10^-5^ | 0.06 | 0.35 |
| DTS | S10_148435462 | T/C | 202/58 | | 2.55 | 7.18 × 10^-5^ | 0.06 | 0.33 |

^a^ The number before the underscore (_) indicates the chromosome number and the number after the underscore (_) indicates the physical position in bp within the chromosome .

^b^ The letter before the diagonal (/) is the nucleotide more frequent ; and the letter after the diagonal the nucleotide less frequent.

^c^ Nº = number of inbred lines homozygous for a determined allelic variant. The number before the diagonal (/) represents the number of individuals with the mayor allele; and the number after the diagonal represents the number of individuals with the minor allele.

^d^ The additive effect was calculated as half the difference between the mean of the homozygous for the minor and the mean of the homozygous for the major allele. It is given in days.

^e^ *R^2^*, proportion of the phenotypic variance explained by the SNP .

^f^ RMIP, resample model inclusion probability.

^g^ Based on SNPs from Illumina chip, the remaining locations without a superscript are based on SNPs obtained by GBS.

**Table S8 Linked disequilibrium (*r^2^*) between the ten SNPs significantly associated to TL made by BMC.**

|  | SNP | [1] | [2] | [3] | [4] | [5] | [6] | [7] | [8] | [9] | [10] |
| --- | --- | --- | --- | --- | --- | --- | --- | --- | --- | --- | --- |
| [1] | S2_168004182 |  | 0.05 | 0.09 | 0.10 | 0.01 | 0.00 | 0.00 | 0.02 | 0.02 | 0.02 |
| [2] | S3_7081859 |  |  | 0.01 | 0.00 | 0.00 | 0.00 | 0.01 | 0.04 | 0.04 | 0.04 |
| [3] | S4_190444179 |  |  |  | **0.91** | 0.00 | 0.00 | 0.01 | 0.08 | 0.08 | 0.08 |
| [4] | ss4_190679094 |  |  |  |  | 0.00 | 0.00 | 0.01 | 0.08 | 0.08 | 0.08 |
| [5] | S7_154739818 |  |  |  |  |  | **0.24** | 0.13 | 0.00 | 0.00 | 0.00 |
| [6] | S7_154741622 |  |  |  |  |  |  | **0.24** | 0.01 | 0.01 | 0.01 |
| [7] | S7_155702328 |  |  |  |  |  |  |  | 0.00 | 0.00 | 0.00 |
| [8] | S10_133337924 |  |  |  |  |  |  |  |  | **1** | **1** |
| [9] | S10_133337925 |  |  |  |  |  |  |  |  |  | **1** |
| [10] | S10_133337950 |  |  |  |  |  |  |  |  |  |  |

SNPs markers with *r^2^* ≥ 0.20 are indicated in bold.
